# A comprehensive immune cycle enhancement strategy for alternative splicing-mediated endogenous Tumor neoantigens generation and delivery

**DOI:** 10.1016/j.mtbio.2025.102231

**Published:** 2025-08-30

**Authors:** Linbang Wang, Yu Liu, Ziyu Wang, Jingkun Liu, Ziqiang Yan, Xiaoguang Liu

**Affiliations:** a Department of Orthopedics, Peking University Third Hospital, Beijing, People's Republic of China; b Department of Orthopedics, Honghui Hospital, Xi'an Jiaotong University, No. 555, Youyi Road, Beilin District, Xi'an, 710054 Shaanxi People's Republic of China

**Keywords:** Osteosarcoma, RNA alternative splicing, BaTiO3 nanocubes, Endogenous Tumor neoantigens, Programmed cell death-1

## Abstract

Although immunotherapy exhibits remarkable clinical potential for the treatment of tumors, immune responses generated by conventional approaches often fail to completely eradicate osteosarcoma. This inadequacy stems primarily from the low immunogenicity of osteosarcoma-derived neoantigens and the limitations of conventional strategies that focus on enhancing only a single step in the tumor immunity cycle and fail to effectively drive a comprehensive immune response. To address these challenges and augment antitumor immune responses, we developed the innovative core-shell nanoparticle system BaTiO3-indisulam@PD1-cell Membrane Nanoparticles (BI@PCM NPs). This system achieves tumor targeting and enables the ultrasound-triggered controlled release of components. Unlike traditional methods that rely on DNA damage-mediated neoantigen production, BI@PCM disrupts alternative RNA splicing, thereby generating high-quality Endogenous Tumor Neoantigens (ETNs). These ETNs are dynamically transported from the tumor site to lymph nodes (LNs) using BaTiO_3_ nanocubes (≈10 nm) as efficient nanocarriers. BaTiO_3_ acts as a piezoelectric catalyst, producing reactive oxygen species (ROS) upon ultrasound stimulation, further enhancing the immunogenic death of osteosarcoma cells. Integration of Pd1 cell membrane coating provides enhanced targeting capabilities and significantly amplifies cytotoxic T-cell activation. By strengthening multiple immune cycle steps, BI@PCM exhibits immense potential to revolutionize personalized tumor immunotherapy and provide a robust solution for osteosarcoma treatment.

## Graphical abstract

**Figure:**
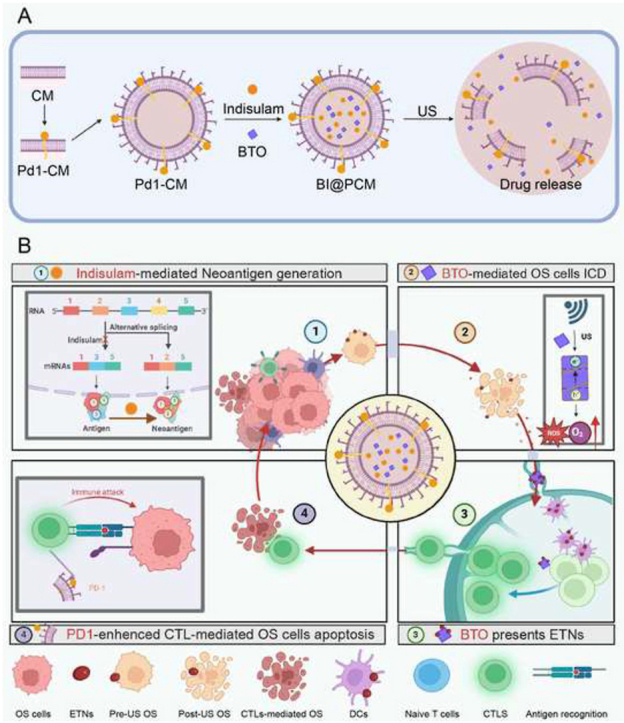

## 1 Introduction

The advent of tumor immunotherapy has profoundly transformed clinical oncology practice. However, the efficacy of current immunotherapeutic agents remains suboptimal, primarily due to their predominant focus on Cytotoxic T Lymphocytes (CTLs) as the sole therapeutic target [1]. CTLs do not function in isolation but are part of the tumor immune cycle, which includes antigen generation, DC-mediated presentation, CTL activation, and tumor cell destruction by effector T cells [2]. Notably, the successful eradication of tumor cells by CTLs triggers a cascade of events, initiating new rounds of antigen presentation and further CTL activation. This sustains an ongoing immune response and allows the immune system to adapt to the evolving tumor microenvironment [3]. However, any stage in this intricate cycle has the potential to become a rate-limiting step, significantly impairing the capacity of the immune system to suppress tumor progression [2]. Currently, the efficacy of traditional therapies combined with immune therapies remains unsatisfactory. The most critical reason for this is the difficulty in fully activating the entire tumor immune cycle, which results in the inadequate overcoming of the immunosuppressive tumor microenvironment (TME). Specifically, although traditional therapies, such as radiation therapy and chemotherapy [4], mediate large-scale immunogenic tumor cell death and release DAMPs [5,6], they fail to fully activate anti-tumor immunity due to issues such as insufficient antigen delivery, and they also cause significant toxic side effects [7,8]. On the other hand, strategies such as tumor vaccines effectively activate tumor immunity but struggle to achieve potent tumor killing [9,10]. Consequently, there is an urgent need for a more comprehensive and integrative approach that enhances the functionality of the entire tumor-immunity cycle. This strategy is crucial for eliciting robust and sustained immune-mediated elimination of tumors.

The generation of tumor antigens represents a pivotal initial step in the tumor immunity cycle [11]. Traditionally, therapeutic modalities such as radiotherapy and chemotherapy have been employed to induce tumor antigen generation by causing DNA damage and promoting genomic mutations, which lead to the production of tumor-specific neoantigens [12]. Despite this strategy, these approaches have exhibited limited effectiveness across various types of tumors [13]. Research has indicated that even in tumors with a high mutational burden such as osteosarcoma, numerous factors hinder the efficient transformation of these mutations into immunogenic neoantigens capable of eliciting robust immune responses [14]. This inability to effectively induce strong immune activation is a key limitation that underlies the poor outcomes associated with such treatments [15]. Interestingly, neoantigen production does not depend exclusively on mechanisms involving DNA damage or mutations. Revisiting the central dogma of molecular biology, which including DNA replication, transcription, translation, highlights that transcriptional diversity at the RNA level is far greater than that at the DNA level [16]. Through intricate transcriptional regulation, a single gene can generate various mRNA isoforms through alternative splicing, thereby significantly expanding the range of possible transcriptional outputs [17]. Recent studies have demonstrated that disrupting alternative splicing of tumor RNA can serve as an alternative mechanism for generating a substantial pool of immunogenic neoantigens [18]. Critically, tumor-specific antigens derived from alternative splicing exhibit greater divergence from self-antigens, characterized by multiple amino acid alterations rather than single-point mutations. This structural distinction likely confers superior immunogenicity compared to mutation-derived neoantigens [19], Specifically, external interventions can trigger incomplete RNA splicing events, leading to the production of intron-derived peptides that are presented by MHC-I molecules on tumor cells to subsequently elicit potent endogenous immune responses. Furthermore, studies demonstrate that tumor mRNA harbors unique exon-exon junctions (neojunctions) absent in normal tissues. These splicing-derived neoantigens are: Expressed in tumor cells, presented on MHC-I complexes and capable of activating T-cell immunity. This paradigm enables the development of precision immunotherapies that selectively target cancer cells while sparing healthy tissues [20], To explore this mechanism, we used indisulam, a small-molecule compound that degrades the RNA splicing factor RBM39 and effectively disrupts the splicing process [21]. Remarkably, indisulam generates ETNs and induces robust immune responses without causing any genomic alterations [22]. This innovative approach offers promising potential for the treatment of immune-resistant Tumors such as osteosarcoma, providing a novel pathway to overcome the limitations of conventional therapeutic strategies.

A significant bottleneck in the Tumor-immunity cycle is the inefficient presentation of tumor antigens, particularly the difficulty in effectively transporting these antigens from the tumor site to draining lymph nodes (DLNs) [2]. Drawing inspiration from studies demonstrating that ultrasmall nanocubes (NCs) can readily traverse stromal barriers and enter the lymphatic system to precisely target DLNs, we introduced the use of highly biocompatible BaTiO_3_ NCs (BTO, approximately 10 nm) as carriers for ETNs. As a piezoelectric material, BTO generates substantial amounts of reactive oxygen species (ROS) that are known for their excellent tissue penetration properties, when stimulated by ultrasound (US) [23]. These ROS not only directly induce the destruction of tumor cells but also significantly enhance their immunogenicity. Importantly, we determined that BTO not only retained its piezoelectric characteristics but also displayed remarkable efficiency for capturing proteins and targeting DLNs with high specificity.

**Scheme 1:**
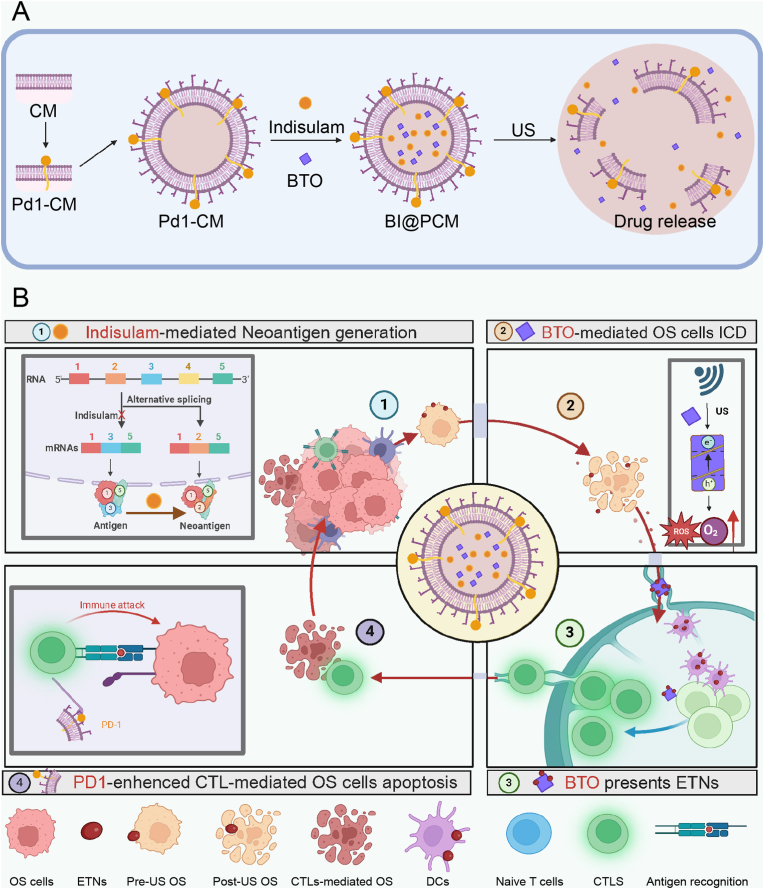
BI@PCM Design and Mechanistic Roles. A. Synthesis and Ultrasound-triggered Release of BI@PCM. This panel illustrates the synthesis process of BaTiO_3_-indisulam nanoparticles encapsulated within a Pd1-expressing cell membrane (BI@PCM). The procedure begins with the overexpression of Pd1 on 293 T cells, followed by membrane extraction to create a biomimetic shell. The final core-shell nanoparticles, BI@PCM, integrate BaTiO_3_ nanoparticles and indisulam, achieving a multifunctional nanoplatforms. Upon exposure to ultrasound (US), the system undergoes structural disintegration, releasing its functional components, including BaTiO_3_ nanoparticles and indisulam, into the tumor microenvironment. B. Mechanistic Contributions of BI@PCM to Enhancing the Tumor-Immunity Cycle. This panel highlights the four ordinal key mechanisms enhanced by BI@PCM in combination with ultrasound. 1. Indisulam-Mediated Neoantigen Generation: the disruption of RNA alternative splicing by indisulam facilitates the production of tumor-specific endogenous neoantigens (ETNs), promoting immune recognition. 2. BTO-Mediated Immunogenic Cell Death (ICD): BaTiO_3_ nanoparticles induce ICD by generating reactive oxygen species (ROS) and releasing damage-associated molecular patterns (DAMPs), thereby stimulating antitumor immune responses. 3. BTO-Mediated ETN Delivery and Presentation: BaTiO_3_ nanoparticles efficiently bind and transport ETNs from the tumor site to lymph nodes (LNs), enhancing antigen presentation to dendritic cells (DCs). 4. Pd1-Enhanced CTL Activation: the Pd1 biomimetic coating promotes cytotoxic T lymphocyte (CTL)-mediated apoptosis of osteosarcoma cells by inhibiting tumor immune evasion through the Pd1/Pd-l1 axis blockade.

To optimize the binding of ETNs by BTO within the tumor microenvironment, we designed a sophisticated core-shell structure. The outer shell consists of cell membranes overexpressing programmed cell death-1 (PD1), whereas the inner core is composed of BTO NCs and small molecules. This structural arrangement ensured that BTO remained encapsulated and shielded from interactions with non-tumor-associated proteins before reaching the tumor site. Upon ultrasound activation, the core-shell structure disintegrates, releasing nanoscale BTO and encapsulating small molecules. These released components intervene at various stages of the immune cycle by disrupting alternative splicing to generate ETNs, inducing immunogenic cell death (ICD), damage-associated molecular pattern (DAMPs) release, and facilitating the release of ETNs. Following their release, the BTO NCs effectively bound to ETNs produced during tumor cell death in the tumor microenvironment. These captured ETNs are subsequently transported to DLNs where they are presented to dendritic cells (DCs), thereby enhancing antigen presentation and advancing the immune cycle. Concurrently, Pd1 on the outer shell interacts with PDL1 expressed on the surface of tumor cells, inhibiting immune evasion mechanisms and promoting T cell recognition and cytotoxic activity (Scheme 1). This comprehensive and integrated strategy synergistically enhances the overall effectiveness of the Tumor-immunity cycle, ultimately achieving more robust and durable antitumor therapeutic outcomes.

## 2 Results and discussion

### 2.1 Synthesis and Characterization of BaTiO_3_ nanocubes

**Fig. 1:**
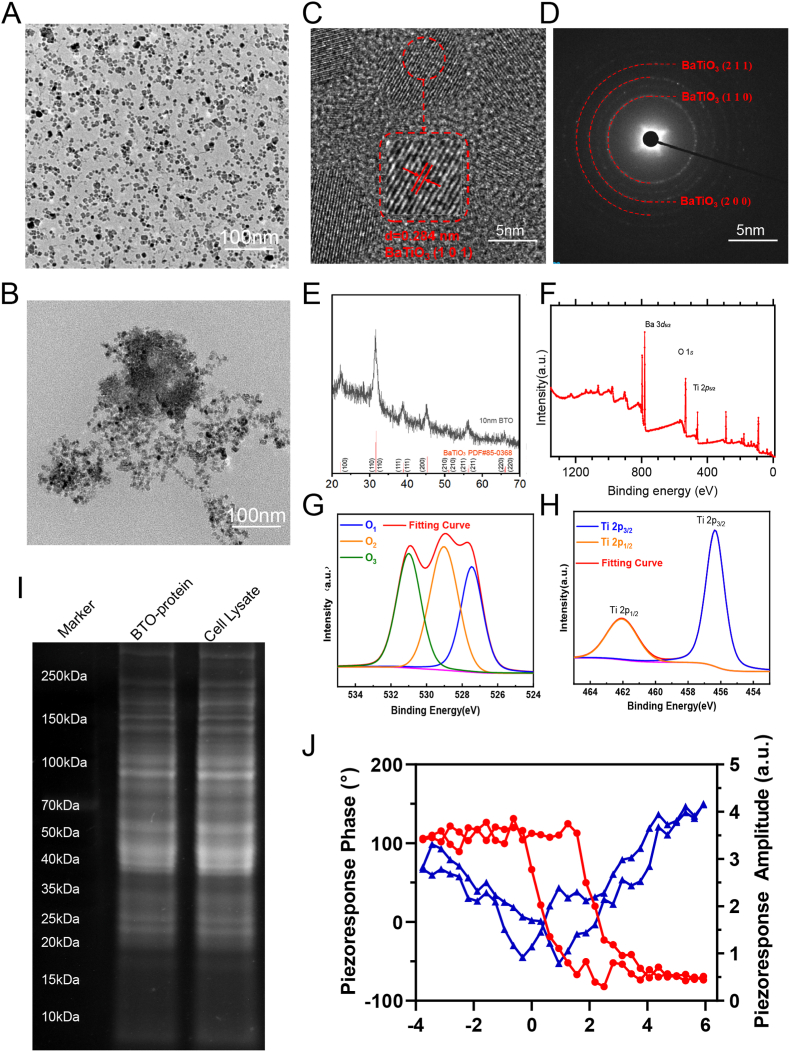
legend Synthesis and Characterization of BaTiO_3_ NCs A. TEM image of as-synthesized BaTiO_3_ nanocubes (BTO NCs). B. TEM image of BTO NCs after incubation with K7M2 tumor cell lysate. C. HRTEM image of BTO NCs. Scale bar, 5 nm. D. Diffraction ring of BTO NCs. Scale bar, 5 nm. E. XRD:XRD pattern of BTO NCs. F. XPS spectra of the BTO NCs. G. Ti 2p spectrum of BTO NCs, H. O 1s spectrum of BTO NCs. I. SDS-PAGE protein analysis of K7M2 cell lysate and BTO NCs-bound protein. Samples were run at equal protein concentrations and were performed with Ultraviolet Imaging. J The PFM phase-voltage and amplitude-voltage loops of the BTO NCs.

The 10 nm BaTiO_3_ nanocubes (BTO NCs) were obtained through a two-phase solvothermal method as previously described [24] (Fig. 1A, SFig. 1). High-resolution transmission electron microscopy (HRTEM) analysis revealed the lattice fringes of a single particle, with an interplanar spacing of d (101) = 2.84 Å (Fig. 1C). The diffraction ring patterns corresponded to the perovskite crystal structure, displaying characteristic diffraction peaks at the (211), (110), and (200) planes (Fig. 1D), indicating a high degree of crystallinity. X-ray diffraction (XRD) further corroborated these findings, exhibiting only peaks consistent with the perovskite phase of the BTO nanocapsules (Fig. 1E), with no additional phases detected. To investigate the elemental composition and chemical states of the BTO NCs, X-ray photoelectron spectroscopy (XPS) was performed. The XPS survey spectra presented in Fig. 1F confirm the presence of barium (Ba), oxygen (O), and titanium (Ti), reflecting the expected composition of the BTO NCs. Additionally, the high-resolution O1s spectra (Fig. 1G) were deconvoluted into three distinct Gaussian peaks, labeled as O1 (527.46 eV), O2 (529.03 eV), and O3 (531.00 eV) that correspond to lattice oxygen (O^2−^) and various surface reactive oxygen species (ROS), including O^2−^−, O_2_^2−^, and O^−^. These observations further highlight the surface characteristics and presence of ROS in the BTO NCs. [25]. The high-resolution Ti 2p XPS spectra of four samples are displayed in Fig. 1H, in which two characteristic binding energy peaks corresponding to Ti^4+^ 2p_3/2_ and 2p_1/2_ were located at 456.34 and 462.05 eV, respectively [25].

BTO NCs effectively captured ETNs, as confirmed by TEM imaging of protein-bound nanoparticles (Fig. 1B), which showed a distinct 'fog' layer. Furthermore, both the size and ζ-potential of the BTO NCs exhibited notable changes post-incubation, providing additional confirmation of protein binding (SFig. 2). The efficiency of ETN capture was further validated by quantifying the total amount of protein associated with the BTO NCs, as presented in SFig. 3. The results indicated a concentration-dependent protein capture by the nanoparticles. Subsequently, to further assess BTO's ability to capture antigenic peptides, we conducted dextran size exclusion chromatography (SEC), where we determined and statistically analyzed the molecular weight of the captured proteins. As shown in SFig. 4, compared to the lysate proteins (pre), the captured proteins (post) exhibited a higher proportion of larger proteins (3–80 kDa). Additionally, peptide segments within the 0.7–1.5 kDa range, which include the 8–14 amino acid long neoantigen peptides, were also substantially captured, confirming BTO's capacity to capture proteins, particularly antigenic peptides, which includes splicing-derived neoantigens validated by proteomics (Table S1) and functional immunogenicity assays. To further investigate the mechanism by which BTO NCs capture endogenous tumor neoantigens (ETNs), we conducted competitive binding assays between BTO NCs and ETNs. The results revealed that increasing spermine concentrations significantly reduced protein adsorption on BTO NCs in a dose-dependent manner (SFig. 5). Building on our prior validation that the captured proteins contain substantial ETNs (Table S1), these findings collectively demonstrate that electrostatic interactions drive ETN capture by BTO NCs. Gel electrophoresis analysis revealed alterations in the protein profile, with a distinct modulation observed when comparing the proteins captured by the BTO NCs to the raw cell lysate (Fig. 1I). Importantly, following protein capture, the BTO NCs were approximately 30 nm in size, and this is optimal for efficient lymph node targeting. Specifically, interstitial flow is a low-resistance transport pathway from tumor tissues to lymph nodes [26,27]. By using very small nanoparticles, this biophysical mechanism can be utilized to drain into the lymph nodes through the interstitial matrix [28]. Several studies have shown that nanoparticles with a size smaller than 30 nm can be effectively transported to draining lymph nodes [29,30]. This feature may be determined by the network structure of the extracellular matrix of the interstitial cells and the overlapping lymphocyte-cell junctions at the valve-like sites [26]. Next,to assess the piezoelectric properties and enzyme-like activity of the BTO NCs, a voltage of 20 V was applied and the oscillation signals of the cantilever were recorded as phase and amplitude signals. The local polarization-electric field loops (P–E) of the BTO NCs exhibit a clear hysteresis loop and 180° phase switching, indicating a robust ferroelectric response. Additionally, the butterfly-shaped amplitude curve further validates the excellent piezoelectric properties of the BTO NCs (Fig. 1J).

As piezoelectric materials, ultrasmall BTO NCs combined with ultrasound can induce imbalanced charges on the surface that react with nearby water molecules to generate ROS and O_2_ [31]. To access the ROS-generating capability of BTO NCs, 2′,7′-dichlorodihydrofluorescein diacetate (DCFH-DA) was used as a probe for its oxidization by ROS, resulting in green fluorescence generation [32]. It was observed that the fluorescence intensity of DCFH-DA was significantly elevated in the BTO + US group. In contrast, neither the BTO-only group (100 μg/mL, BTO NCs) nor the US-only group (1 MHz, 1 W/cm^2^, 50 % duty cycle) exhibited any substantial change in DCFH-DA fluorescence relative to that of the PBS group (SFig. 6). These findings indicate that only the combined application of BTO NCs and ultrasound (US) effectively mediated the piezocatalytic reaction to generate reactive oxygen species (ROS), with the cell membrane coating exerting minimal influence on the piezocatalytic process. Additionally, the O_2_ generation from BTO NCs dispersed in deoxygenated deionized water was evaluated, yielding results consistent with those of the ROS generation experiment. The O_2_ production exhibited a clear dependence on both the concentration of BTO NCs and the duration of ultrasound irradiation, notably, O_2_ was exclusively produced in the BTO NCs + US group (SFig. 7), further confirming the piezocatalytic capacity of BTO NCs to catalyze water splitting, independent of the tumor microenvironment.

### 2.2 Construction of BaTiO3-indisulam@PD1-cell membrane nanoparticles and ultrasound-induced release

**Fig. 2:**
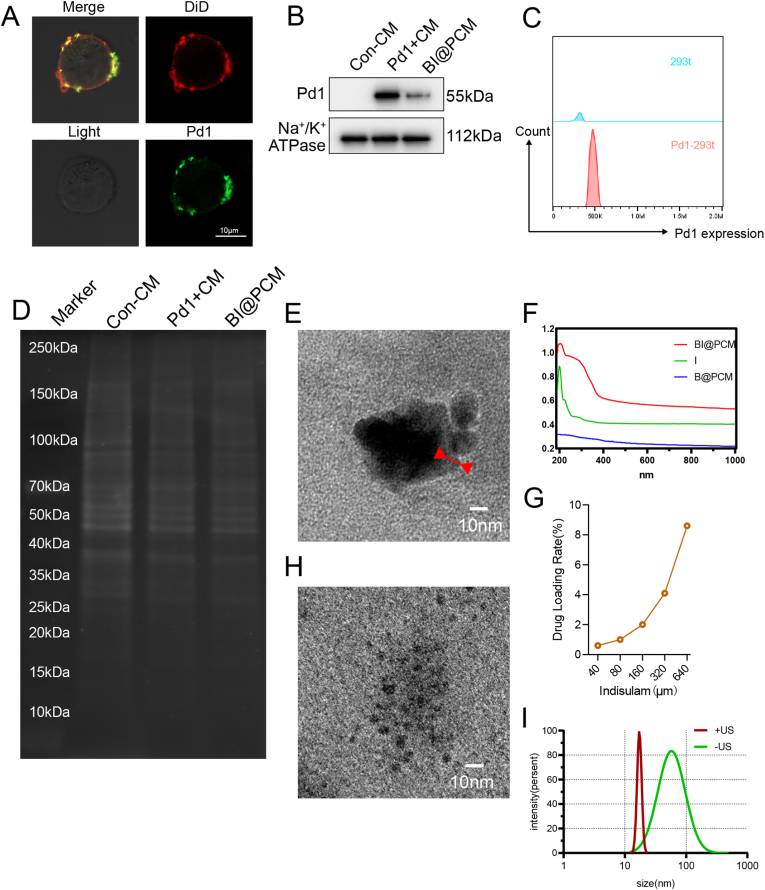
legend Construction and Ultrasound-Induced Release of BI@PCM Nanoparticles A. Fluorescence microscopy of 293 T-Pd1 cells (GFP-tagged Pd1 localization. B. Western blot (WB) analysis of membrane protein expression in Pd1-overexpressing cells compared to that of the control group. C. Flow cytometry results illustrating the differences in Pd1 expression levels between Pd1-overexpressing and control cells. D. SDS-PAGE analysis indicating the membrane protein profiles across different experimental groups. E. TEM of BI@PCM nanoparticles before ultrasound treatment. F. UV–vis absorbance spectra illustrating the drug-loading efficiency of BI@PCM. G. Drug-loading rate analysis of BI@PCM at varying concentrations, providing quantitative insight into loading efficiency. H. TEM images highlighting the structural disintegration of BI@PCM after US treatment (US: 1 MHz, 1.5W/cm^2^, 30 % duty cycle). I. Particle size distribution of BI@PCM nanoparticles before and after US treatment, indicating significant size reduction.

To achieve optimal protein-binding efficiency of BTO NCs within tumor tissues, we engineered and synthesized a core-shell nanoparticle structure comprising BTO NCs and indisulam at the core and encapsulated by a Pd1 cell membrane (PD1-CM) shell that we designated as BaTiO_3_-indisulam@PD1-cell membrane Nanoparticles (BI@PCM NPs, Scheme 1A). The Pd1/Pd-l1 signaling axis plays a pivotal role in modulating tumor immunity. Specifically, Pd-l1 overexpressed in tumor cells interacts with Pd1 on cytotoxic T lymphocytes (CTLs), thereby inhibiting their activation and facilitating immune evasion. Consequently, blocking of the Pd1/Pd-l1 immune checkpoint has emerged as a crucial strategy for counteracting the immunosuppressive microenvironment in osteosarcoma, thereby enhancing the capacity of the immune system to target and eradicate tumor cells. [33]. *In situ* engineering for Pd1 overexpression on the cell membrane, as opposed to conventional chemical coupling methods, can significantly enhance the localized activity of Pd1. This approach facilitates the competitive binding of Pd1 to Pd-l1, thereby more effectively disrupting the Pd1/Pd-l1 signaling axis. This strategy optimizes the inhibition of this immune checkpoint, promoting a stronger and more targeted immune response against tumor cells [34].In this study, we employed a genetic engineering approach to achieve stable *in situ* Pd1 overexpression in 293 T cells. These 293 T cells stably expressing Pd1 were generated by transduction with the lentiviral vector [LV2 CMV-EF1α-cGFP(2 A)-Puro] that encodes the Pd1 gene (SFig. 8). After lentiviral infection, cells were tagged with green fluorescent protein (GFP) and selected using puromycin. Fluorescence microscopy further revealed GFP localization to the 293 T cell membrane (Fig. 2A), with highly increase in the Pd1 fluorescence signal, indicating successful upregulation of Pd1 expression in 293 T-Pd1 cells. Additionally, flow cytometry (FCM) and western blot (WB) analysis confirmed the overexpression of Pd1 (Fig. 2B and C). Gel electrophoresis analysis revealed alterations in the membrane protein profile (Fig. 2D). These results collectively validate the successful establishment of the Pd1 overexpressing 293 T-Pd1 cell line. Next, to further demonstrate that PD1 can bind to PD-L1 and exert its biological function, we conducted a Co-IP experiment to characterize the protein-protein interaction. The results confirmed that the synthesized PD1 can indeed bind to PD-L1 (SFig. 9).

Transmission electron microscopy (TEM) images (Fig. 2E) clearly illustrate that the BTO nanocapsules (NCs) possess a core encapsulated by a lipid bilayer derived from the cell membrane, as indicated by the red arrows. This lipid bilayer serves as a protective barrier for the BTO core, effectively preventing the uptake of non-tumor-associated proteins during systemic circulation. Fourier transform infrared (FT-IR) spectroscopy further supports the presence of the engineered cellular membrane on the BTO NCs, revealing characteristic absorption peaks around 550 cm^−1^ corresponding to the BTO core, as well as distinct bands associated with the cell membrane (SFig. 10), Specifically, the bands at around 550 cm-1 were due to the size effect influencing the Ti-O stretching mode of BaTiO3 [35,36]. This distinct characteristic peak was also observed after coating with the cell membrane. Additionally, the integrity of the membrane proteins was maintained throughout the gene engineering, extraction, and coating processes applied to the BTO NCs, as evidenced by the data presented in Fig. 2D. The drug-loading efficiency of the BI@PCM nanoparticles for indisulam was assessed using UV–vis absorbance spectra that indicated a drug-loading capacity of approximately 6 % by the characteristic peak of Indisulam observed at ≈199 nm and was calculated by the following formula: $\text{Drug}\;\text{Loading}\;\text{Rate}\;\left(\%\right)=100*\frac{I\text{ndisulam}\;\text{weight}\;\text{inside}\;\text{the}\;\text{NPs}}{\text{Total}\;\text{weight}\;\text{of}\;\text{the}\;\text{NPs}}$ (Fig. 2F and G, SFig. 11). It is suggested that Indisulam is predominantly physically entrapped within the nanomedicine formulation. Next, loading stability assessments of Indisulam demonstrate that 50 % retention of Indisulam was maintained after 7 days of storage in PBS at 25 °C (compared to initial loading, SFig. 12). Notably, the release of ultrafine BTO NCs within tumor tissues is essential, as their nanoscale size is crucial for facilitating efficient lymph node targeting. TEM analysis further demonstrated the successful release of BTO-containing components from the BI@PCM particles upon ultrasound (US) treatment (Fig. 2H), likely due to ROS-mediated disruption of the lipid bilayer shell that allows for controlled drug release. The average hydrodynamic diameter of the BI@PCM nanoparticles underwent a significant reduction from 175 nm to approximately 7 nm following ultrasound (US) treatment, as revealed by the size distribution analyses (Fig. 2I), this change provides further evidence for the successful release of BaTiO_3_ NCs.

Furthermore, Importantly, the release profile of indisulam also demonstrated a marked increase post-US stimulation, exhibiting a notable enhancement compared to the conditions prior to US exposure. The concurrent release of indisulam and BTO was distinctly observed (SFig. 13), underscoring the synergistic effect of the US-induced release mechanisms. This dual-release process not only validates the efficacy of the US-triggered system but also highlights the potential for targeted and controlled therapeutic delivery. The optimization of ultrasound parameters (power, frequency, duration) is critical for balancing the release kinetics of BTO/indisulam and ROS-mediated cytotoxicity. Based on systematic screening (SFig. 14), we selected 1.5 W/cm^2^, 1 MHz, 100 s duration, and 30 % duty cycle as the optimal condition. This setting achieves fully release of (>80 % within 100 s) Our parameter optimization reveals a fundamental trade-off: mild ultrasound favors controlled drug release, while intense fields maximize piezoelectric cytotoxicity. The 1.5 W/cm2 compromise ensures both effective Indisulam deployment and ROS generation. Future work exploring spatiotemporally modulated ultrasound (e.g., dual-frequency sequences) may further decouple these effects, potentially enhancing tumor-specific activity while reducing off-target damage.

To further investigate whether PD1-cell membrane coating affects BTO's piezoelectric performance, we conducted time-resolved measurements of ROS and O_2_ generation. As shown in Supplementary SFig. 15 and SFig. 16: Before 200s: Membrane-coated BTO NCs exhibited lower ROS/O_2_ production than uncoated BTO NCs. After 200s: Membrane-coated BTO NCs achieved 100 % of the ROS/O_2_ levels observed in uncoated BTO NCs. The membrane coating initially attenuates piezoelectric activity but, following ultrasound-induced structural rupture, allows complete restoration of BTO's piezoelectric function. This ensures sustained therapeutic efficacy both in vitroand in vivo.

### 2.3 BI@PCM combined with US mediates tumor ICD and DAMP release

**Fig. 3:**
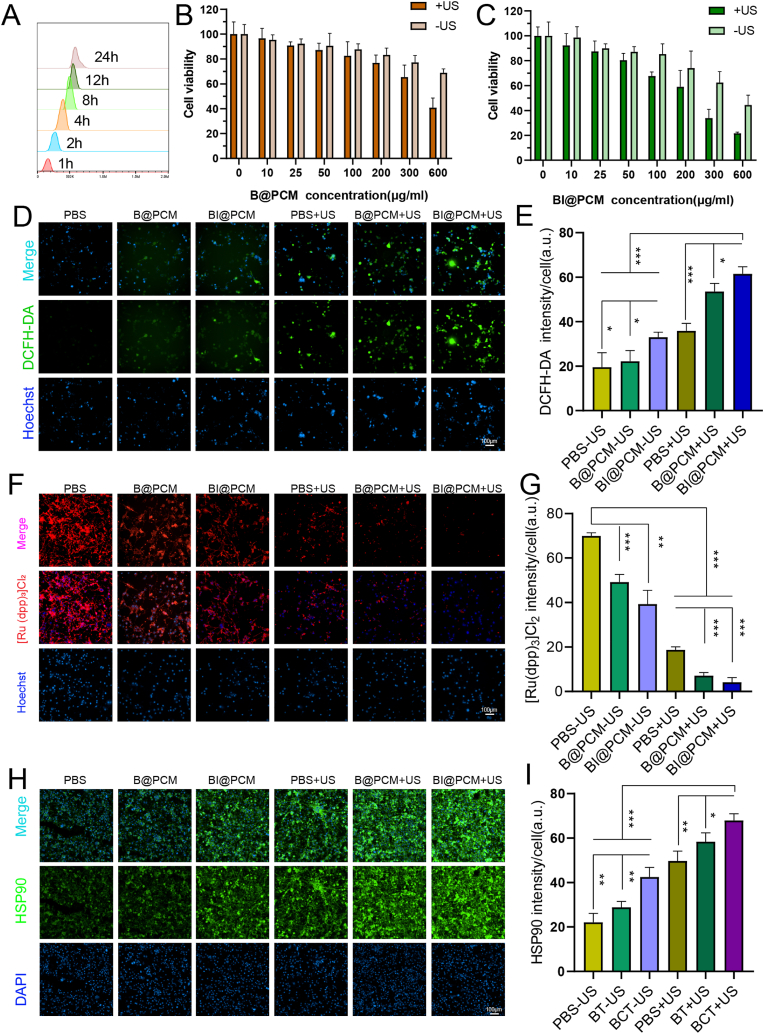
legend BI@PCM Combined with US Mediates ICD and DAMP Release (A) Flow cytometry analysis indicating the phagocytosis of BI@PCM by K7M2 cells. (B) Cytotoxicity of B@PCM at different concentrations under -US and +US conditions (US: 1 MHz, 1.5W/cm^2^, 30 % duty cycle, 100s). (C) Cytotoxicity of BI@PCM at different concentrations under -US and +US conditions. (D) Total ROS production in cells across groups, including PBS-US, B@PCM-US, BI@PCM-US, PBS + US, B@PCM + US, and BI@PCM + US (dosage: 200 μg/mL). (E) Quantification of fluorescence intensity for ROS (n = 3, mean ± SD). (F) Intracellular O_2_ levels across groups, including PBS-US, B@PCM-US, BI@PCM-US, PBS + US, B@PCM + US, and BI@PCM + US. (G) Quantification of fluorescence intensity for Ca^2+^ (n = 3, mean ± SD). (H) Immunofluorescence detection of HSP90 across groups, including PBS-US, B@PCM-US, BI@PCM-US, PBS + US, B@PCM + US, and BI@PCM + US. (I) Quantitative analysis of HSP90 fluorescence intensity across groups, including PBS-US, B@PCM-US, BI@PCM-US, PBS + US, B@PCM + US, and BI@PCM + US.

We initially assessed the uptake of BI@PCM NPs by K7M2 cells at multiple time points (Fig. 3A). The data revealed a rapid and substantial internalization of the nanoparticles within 4 h, and this was maintained for over 12 h, demonstrating the prolonged presence of BI@PCM NPs within the cells. SFig. 17 presents flow cytometry analysis comparing the uptake of BI@PCM NPs (PD1-high membrane) versus B@CM NPs (PD1-low membrane) in K7M2 cells at 4 h. SFig. 18 quantitatively demonstrates the time-dependent uptake kinetics of BI@PCM NPs and B@CM NPs in K7M2 cells at 1 h, 2 h, 4 h, 8 h, 12 h, and 24 h. Results indicate that K7M2 cells exhibit significantly higher uptake efficiency for BI@PCM NPs (2.1-fold increase at 4 h; p < 0.001) compared to B@CM NPs, confirming the critical role of PD1/PD-L1 interaction in targeted delivery via competitive binding to PD-L1. Furthermore, building on prior reports that PD1-cell membrane coating enhances tumor-targeted delivery, we systematically compared the cellular uptake of variously coated nanoparticles in tumor cells (K7M2 cell line) versus normal cells (NIH/3T3 cell line) relative to uncoated BTO or membranes without PD1 overexpression. The results demonstrate significantly higher uptake in tumor cells compared to normal cells for nanoparticles coated with PD1-overexpressing membranes. These data conclusively demonstrate that PD1 overexpression on membranes drives active tumor targeting via PD1/PD-L1 ligand-receptor interaction. Subsequently, we investigated the cytotoxic effects of the NPs at different concentrations and ultrasound (US) conditions ( ± US). The CCK-8 assay results indicated a clear dose-dependent increase in cytotoxicity in all experimental groups. Notably, BI@PCM NPs exhibited significantly enhanced tumor cell lethality compared to that of B@PCM NPs, underscoring the potent antitumor activity of indisulam, a sulfonamide-based therapeutic agent, when incorporated into the nanoparticle formulation. This finding suggests that the synergistic effect of BI@PCM NPs and ultrasound (US) can effectively enhance the therapeutic efficacy against osteosarcoma cells [37]. Furthermore, US stimulation significantly augmented the inhibition of cell proliferation. Notably, BTO NCs exhibited a marked increase in cytotoxicity at concentrations exceeding 200 μg/mL under exogenous US exposure. In the B@PCM + US group, the survival rate of K7M2 tumor cells decreased to approximately 50 %, whereas in the BI@PCM + US group, it further decreased to approximately 30 % (Fig. 3B and C), highlighting the enhanced antitumor effect induced by the combination of BI@PCM NPs and US.

Considering the ROS and O_2_ generation capabilities of BTO, we used the fluorescent probe 2,7-dichlorodihydrofluorescein diacetate (DCFH-DA) to quantify the intracellular ROS levels after each treatment. As illustrated in Fig. 3D and E, the BI@PCM group displayed significantly more intense fluorescence under + US conditions, suggesting that US stimulation notably increased ROS production within cells, and this was mediated by BI@PCM. To further validate this result, we also stained the cells in each group with Hydroxyphenyl fluorescein (HRF) and captured the fluorescence of both DCFH-DA and HRF-stained cells using high magnification. The results were consistent with the previous findings (SFigs. 19–22). To further investigate the O_2_ production efficiency of each nanoparticle formulation, we employed the oxygen-sensitive probe [Ru (dpp)_3_]Cl_2_. The results demonstrated that the BI@PCM nanoparticles produced a significantly higher fluorescence intensity than did B@PCM and PBS, with a marked enhancement observed under + US conditions (Fig. 3F and G). This finding unequivocally confirms that US stimulation effectively catalyzes the generation of O_2_, further potentiating the therapeutic efficacy of BI@PCM.

To further assess the effect of BI@PCM nanoparticles on cell proliferation, we employed calcein-AM/propidium iodide (PI) co-staining followed by confocal microscopy to visualize live and dead cells under both + US and -US conditions. Fluorescence imaging revealed a marked increase in tumor cell death in the BI@PCM + US group, indicating a potent tumoricidal effect facilitated by the combination of BI@PCM and ultrasound treatment (SFigs. 23 and 24).

Additionally, we sought to determine whether this therapeutic approach could induce immunogenic cell death (ICD) in osteosarcoma cells, thereby triggering immune activation and enhancing immune-mediated tumor elimination. To test this hypothesis, we evaluated the release of damage-associated molecular patterns (DAMPs) critical for immune activation. Immunofluorescence staining of HSP90, a well-known marker of ICD, revealed that BI@PCM + US treatment induced the most pronounced ICD response among all experimental groups (Fig. 3H and I). Furthermore, enzyme-linked immunosorbent assay (ELISA) confirmed that treatment with BI@PCM + US significantly elevated the release of HMGB1, a pivotal DAMP molecule, compared to the other treatment groups (SFig. 25). These findings collectively suggest that BI@PCM nanoparticles, in conjunction with US, not only exert direct cytotoxic effects but also facilitate the release of key DAMPs, thereby promoting immune activation and potentially enhancing antitumor immunity.

**Fig. 4:**
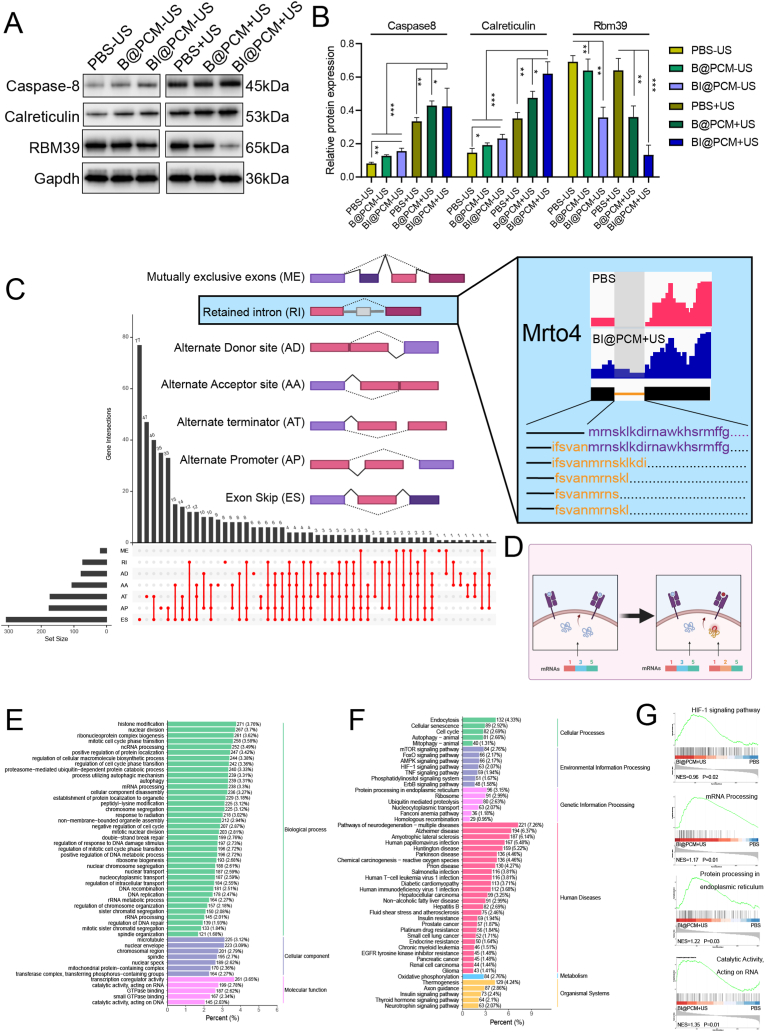
Legend Alternative Splicing Disruption and Neoantigen Generation by BI@PCM + US A. Western blot (WB) bands for caspase-8, CRT, Rbm39, and Gapdh across different treatment groups, including PBS−US, B@PCM−US, BI@PCM−US, PBS + US, B@PCM + US, and BI@PCM + US. B. Quantitative analysis of WB results for caspase-8, CRT, Rbm39, and Gapdh across the same groups. C. Left: Upset plot depicting the statistics of alternative splicing variations and schematic representation of splicing subtypes, where the gray one represent introns. Right: Transcript read coverage for Mrto4 retained introns. D. Schematic diagram of interfering with alternative splicing to enhance tumor cell immunogenicity. The left side shows the antigens presented by MHC II before interference, while the right side shows the diversity of antigens presented by MHC II after interference, E. GSEA-based GO enrichment analysis. F. GSEA-based KEGG enrichment analysis. G. GSEA enrichment pathway visualization.

To investigate the mechanism underlying ICD induction by BI@PCM + US, we assessed the expression levels of key markers, including caspase-8 and calreticulin (CRT), in treated osteosarcoma cells. Caspase-8, a crucial cysteine aspartic protease, is instrumental for the initiation of immunogenic cell death. This facilitates the translocation of CRT from the cytosol to the cell surface, where it functions as an "eat me' signal, thereby promoting the recognition and phagocytosis of dying cells by antigen-presenting cells (APCs). This process is central to the immune activation associated with ICD, as the exposure of the cell membrane to CRT triggers the uptake of tumor cells by APCs and enhances subsequent immune responses. By examining the expression and localization of these markers, we sought to elucidate the specific molecular events involved in the immunostimulatory effects of BI@PCM + US [38]. Western blot analysis indicated that caspase-8 and CRT were significantly upregulated in the BI@PCM + US group, with expression levels markedly higher in the +US group compared to those in the -US group (Fig. 4A and B). These findings suggest that BI@PCM combined with US induces ICD in tumor cells via the caspase-8-CRT pathway.

### 2.4 Effects of BI@PCM combined with ultrasound on alternative splicing disruption and neoantigen generation

We explored the effects of BI@PCM + US treatment on the disruption of alternative splicing and neoantigen generation. Indisulam, a pivotal component of the BI@PCM formulation, functioned as a molecular glue degrader. It facilitates aberrant interactions between RBM39, a key regulator of alternative splicing, and E3 ubiquitin ligase complexes. This interaction results in the targeted degradation of RBM39, thereby impairing normal alternative splicing machinery. Consequently, the disruption of alternative splicing processes not only alters the expression of various splicing isoforms but also contributes to the generation of neoantigens. These neoantigens, arising from abnormal splicing events, can be presented by a major histocompatibility complex (MHC) on the surface of tumor cells, further enhancing immune recognition and potentially stimulating antitumor immune responses [39]. To confirm this observation, we initially examined RBM39 expression levels in K7M2 cells under various treatment conditions using western blot (WB) analysis. The findings revealed a marked decrease in RBM39 expression in both the BI@PCM and BI@PCM + US treatment groups, demonstrating that indisulam effectively facilitated RBM39 degradation (Fig. 4A and B).

To investigate the disruption of alternative splicing and the potential generation of neoantigens associated with tumor immunity in greater detail, we conducted high-throughput RNA sequencing (RNA-seq) of K7M2 cells treated with BI@PCM + US and PBS. This allowed us to quantify the differential expression of genes associated with the various alternative splicing subtypes (SFig. 26). Given that tumor immunity is highly dependent on the antigen presentation pathway mediated by MHC class I molecules, these analyses provided insights into the underlying mechanisms involved [40]. We specifically analyzed all 8–14 amino acid sequences (8-14-mers) generated by alternative splicing events [41]. Utilizing the NetMHCPan algorithm, we evaluated the binding affinities of these sequences to the prevalent MHC I alleles and identified 442 neoantigenic peptides ranging from 8 to 14 amino acids in length and exhibiting significant binding potential to MHC I. These findings confirmed that BI@PCM + US treatment induced profound alterations in both alternative and constitutive splicing patterns. Among these, exon skipping, alternative promoter and retained introns were most commonly observed (Fig. 4C, left). The BI@PCM + US treatment also resulted in a significant induction of retained introns (SFig. 27). As illustrated in Fig. 4C (right), transcript read coverage analysis of Mrto4 revealed the presence of multiple retained intron sequences (Fig. 4D). Notably, retained introns have been recognized for their exceptionally high immunogenic potential among various splicing subtypes [42]. Building upon the observations from transcriptome sequencing, we further conducted RT-PCR experiments. From the top three alternative splicing events—Alternate Promoter (AP), Exon Skip (ES), and Retained Intron (RI)—we selected one representative gene from each event to validate the expression of the indisulam-mediated alternative splicing neoantigen-related mRNAs observed in the transcriptome analysis. Specifically, we analyzed Mrto4 (representing the RI event), Anapc1 (representing the AP event), and Clk4 (representing the ES event). The results, as shown in SFig. 28, indicate that the expression of the relevant mRNAs significantly increased after interference, which is consistent with the transcriptome sequencing results. These results provide compelling evidence that the BI@PCM + US approach effectively promotes RBM39 degradation, and this in turn facilitates the generation of tumor-associated neoantigens, thereby contributing to the disruption of splicing fidelity and enhancement of tumor immunogenicity.

Furthermore, to further confirm the generation of peptides mediated by alternative splicing disruption, we conducted proteomics assessments. Specifically, we compared the 422 variable splicing antigenic peptides predicted from the transcriptome with theoretical cleavage peptides from the normal protein database. We then screened and identified the unique peptides of variable splicing and rebuilt a database. Subsequently, we searched for these unique peptides in the protein mass spectrometry data from the samples and conducted quantitative evaluations. The results revealed the expression of 47 peptides (Table S1). Additionally, by analyzing their MHCI binding scores, we confirmed that these peptides have the potential to act as tumor immunogenic antigen peptides. To analyze the characteristics of these captured antigenic peptides, we used the Cello online tool (http://cello.life.nctu.edu.tw/) to evaluate the cellular localization features of these peptides. The results are also presented in Table S1. Interestingly, we found that these peptides were predominantly predicted to localize extracellularly, followed by mitochondrial and nuclear localization (SFig. 29). We suggest that this is closely related to the EPR effect, surface potential, and size limitations (∼9 nm) of BaTiO_3_.

To investigate the broader biological impacts of BI@PCM + US treatment, we performed Gene Set Enrichment Analysis (GSEA) on the group devided in BI@PCM + US treatment group and PBS group. Unlike traditional enrichment approaches, GSEA utilizes the ranking of genes rather than solely depending on predefined significance thresholds. This methodology enables the identification of nuanced biological effects, detection of weak signals, and characterization of intricate biological processes, thereby providing a more comprehensive understanding of the molecular changes elicited by treatment [43]. The GSEA results highlighted significant disruptions in several KEGG pathways, including the HIF-1 signaling pathway and protein processing within the endoplasmic reticulum (ER) along with notable changes in various GO terms such as mRNA processing and catalytic activity targeting RNA (Fig. 4E–G). The observed alterations in the HIF-1 signaling pathway that governs cellular adaptation to hypoxic conditions were likely influenced by the oxygen-generating properties of BI@PCM + US treatment. Changes in ER protein processing are strongly linked to ER stress responses, with associated genes potentially producing distinct protein isoforms via alternative splicing, thereby modulating cellular stress adaptation mechanisms [44]. Specifically, the accumulation of misfolded/unfolded proteins in the ER lumen triggers ER stress and activates the unfolded protein response (UPR). UPR primarily functions through three signaling proteins: Inositol-requiring enzyme-1α (IRE1α), Protein kinase RNA-like ER kinase (PERK), and Activating transcription factor 6 (ATF6). IRE1α is a transmembrane protein with both Ser/Thr kinase and RNase activities. The accumulation of unfolded proteins in the ER lumen induces dimerization of IRE1α in the ER membrane, which activates its RNase activity through autophosphorylation. The RNase activity of IRE1α catalyzes the splicing of mRNAs, including XBP1, and enhances the transcription of ER chaperone genes, contributing to biological functions such as cell survival and protein homeostasis [45].We quantitatively analyzed the splicing variants of the XBP1 gene by qPCR. The results showed that after treatment, the mRNA level of XBP1(s) significantly increased, while the level of XBP1(u) significantly decreased, thus alleviating ER stress (SFig. 30). Alterations in mRNA processing and RNA-related catalytic activity, both of which are closely associated with the regulation of alternative splicing, were also evident, and this was consistent with the anticipated outcomes. These observations underscore the far-reaching biological effects of BI@PCM + US therapy, particularly the pronounced disruption of alternative splicing mechanisms. Together, these effects play a pivotal role in enhancing tumor immunogenicity, further illustrating the therapeutic potential of this strategy.

To further validate the immunogenicity of splicing-generated peptides, we performed co-culture experiments, where the nanoparticle system, after capturing proteins, was co-cultured with DC cells. The results showed that the expression of the DC surface molecule CD86 was significantly upregulated (SFigs. 31 and 32). Furthermore, ELISA results demonstrated that after the nanoparticle system, which captured proteins, interacted with DC cells, the secretion level of the cytokine IL-12 was significantly increased (SFig. 33). We then co-cultured this system with mouse spleen lymphocytes to observe its effect on T cell activation. The results indicated that compared to the system without splicing-generated peptides (i.e., PBS and the group without indisulam treatment), the co-culture system containing splicing-generated peptides significantly activated spleen lymphocytes. Specifically, the expression of the CD69 surface molecule on spleen lymphocytes was significantly upregulated (SFig. 34, SFig. 35), and the TNF-β levels in spleen lymphocytes were significantly increased (SFig. 36). In a sum, BI@PCM + US therapy preferentially enrich 0.7–1.5 kDa peptides (SFig. 4), including 47 splicing-derived ETNs identified by proteomics (Table S1) that elicit potent immune activation (SFigs. 31–36).

### 2.5 Evaluation of BI@PCM + US in enhancing the tumor immunity cycle using an organoid-immune microenvironment model

This organoid-based drug sensitivity assay integrated with autologous Peripheral blood mononuclear cells (PBMCs) offers a unique combination of advantages [46]. Organoids retain the essential features of the original tumor, including their histological and molecular characteristics [47], while faithfully replicating the complexity of the tumor immune microenvironment. This innovative approach enables the dynamic evaluation of immune-tumor interactions in a physiologically relevant context. Its high degree of clinical relevance makes it a powerful tool for advancing drug discovery and development as well as for guiding personalized therapeutic strategies tailored to individual patients [48]. In this study, we aimed to use this system to evaluate whether BI@PCM + US could enhance the tumor immunity cycle in a personalized manner and improve the efficacy of immunotherapy in human patients.

**Fig. 5:**
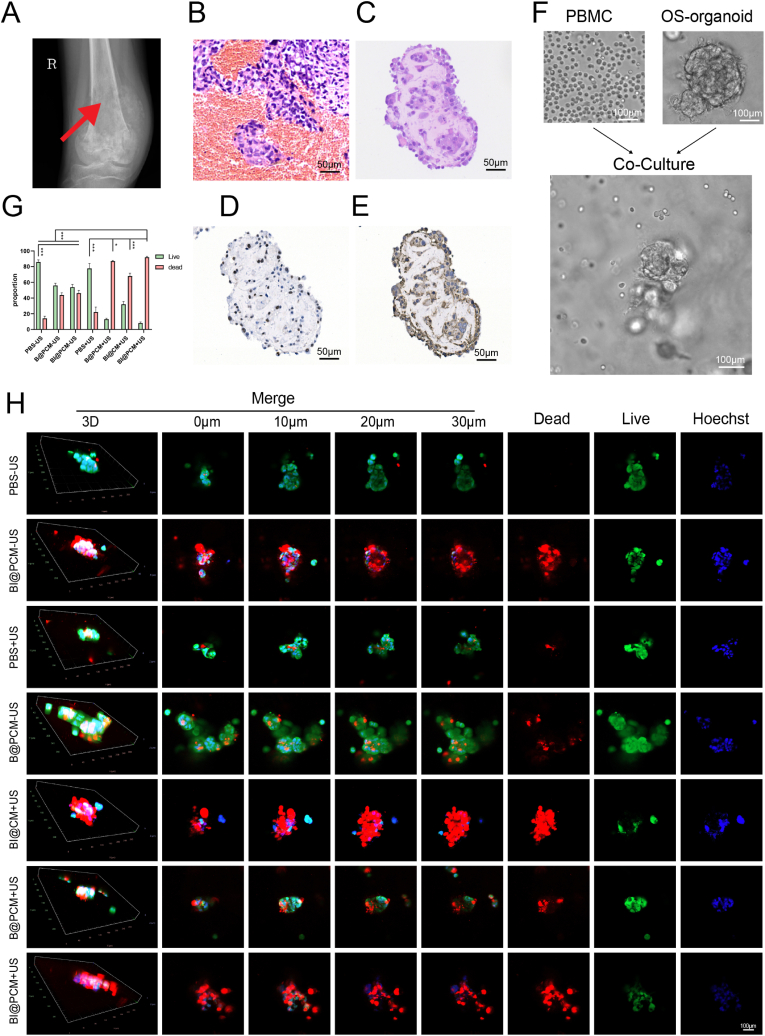
Legend Organoid-PBMC Model for BI@PCM + US Tumor Immunity Enhancement A. X-ray image of the osteosarcoma region before surgery. B. Hematoxylin and eosin (HE) staining of the surgically resected osteosarcoma tissue. C. HE staining of the osteosarcoma-derived organoid. D. Immunohistochemical analysis of Ki67 expression in the osteosarcoma organoid. E. Immunohistochemical analysis of Gapdh expression in the osteosarcoma organoid. F. Workflow diagram illustrating the co-culture of the osteosarcoma organoid with PBMCs. G. Quantitative analysis of live-dead staining in organoid-PBMC systems across different treatment groups, including PBS, PBS + US, B@CM, B@CM + US, BI@CM + US, B@PCM + US, and BI@PCM + US. H. Representative live-dead staining images of organoid-PBMC systems in the respective treatment groups, 0 μm, 10 μm, 20 μm, and 30 μm at the top of the image represent the depth of the Z-axis during confocal shooting.

Organoids derived from a patient with osteosarcoma were successfully established (Fig. 5A), developing within four weeks and maintaining their viability for up to five weeks. Histological examination confirmed the successful construction of the organoids, revealing a morphology that closely mirrored the patient's original tumor tissue (Fig. 5B and C). Additionally, immunohistochemical staining revealed robust Ki67 expression (Fig. 5D), compared with Gapdh (Fig. 5E), providing strong evidence of organoid viability and active proliferative capacity. These findings underscore the effectiveness of this approach for generating patient-specific tumor models for further investigation.

As presented in the workflow outlined in Fig. 5F, organoids were co-cultured with autologous PBMCs from the same patient to replicate the immune microenvironment [49]. The organoids were subsequently allocated to various treatment groups, including PBS, PBS + US, B@CM, B@CM + US, BI@CM + US, B@PCM + US, and BI@PCM + US (Among them, PCM represents the cell membrane with high Pd1 expression, and CM represents the cell membrane with low Pd1 expression). The live-to-dead cell ratio within the organoids was evaluated after treatment to thoroughly assess the therapeutic efficacy of the combination strategy. The analysis revealed that the BI@PCM + US group possessed the highest proportion of dead cells (Fig. 5G and H), outperforming all other groups, with the B@PCM + US and BI@CM + US groups exhibiting the next most pronounced effects. These findings highlight the superior tumor-killing capability of the BI@PCM + US approach compared to that of alternative treatment regimens.

### 2.6 Distribution, lymph node targeting, and antitumor efficacy of BI@PCM combined with ultrasound in tumor-bearing mice

**Fig. 6:**
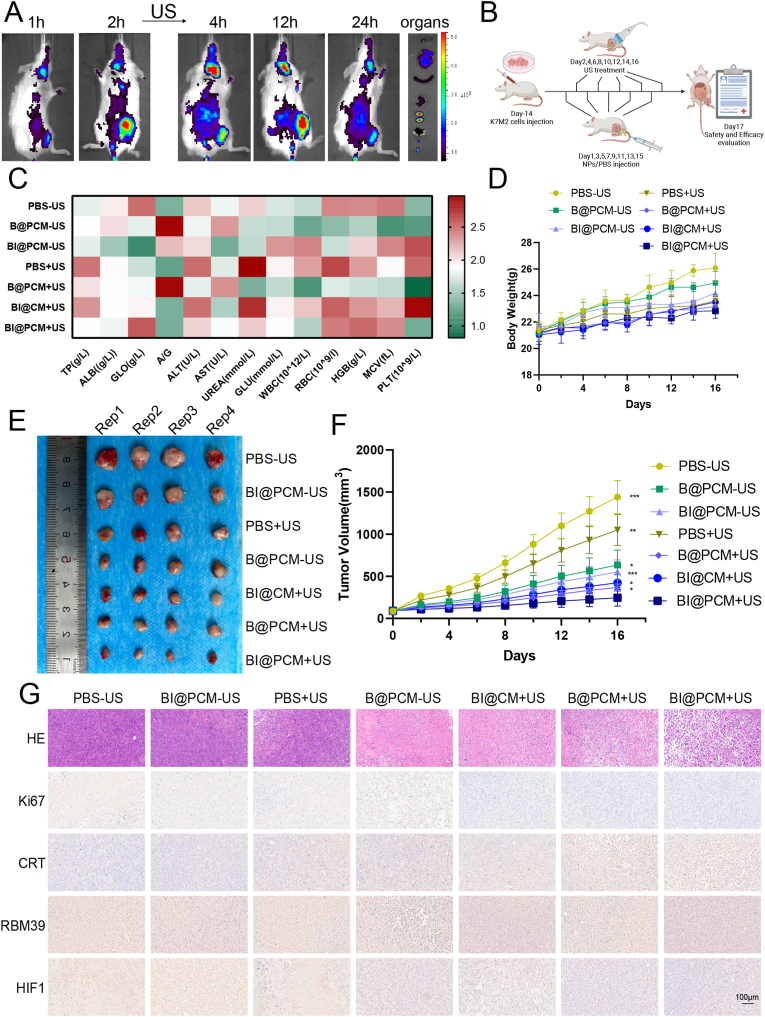
Legend Distribution, Lymph Node Targeting, and Antitumor Efficacy of BI@PCM + US A. *In vivo* fluorescence imaging of K7M2 tumor-bearing mice at various time points after tail vein injection of Cy5.5-labeled BI@PCM, with and without ultrasound treatment, along with *ex vivo* fluorescence imaging of major organs and tumor tissues. B. Schematic representation of the mouse treatment regimen. C. Changes in body weight over time across different treatment groups. D. Analysis of peripheral blood biochemistry and complete blood counts post-treatment. E. Representative gross images of tumors at the end of treatment (dosage: 2 μg/g. US: 1 MHz, 1.5W/cm^2^, 30 % duty cycle, 100s). F. Tumor volume changes over time across different treatment groups. G. Histological staining of tumor tissues following treatment in each group.

Encouraged by the exceptional efficiency of BTO for capturing ETNs, we investigated whether the final BI@PCM formulation could actively transport ETNs from ultrasound-treated tumor sites to lymph nodes (LNs). To monitor the in vivo biodistribution of BI@PCM, BTO was labeled with Cy5.5 and administered via tail vein injection into K7M2 tumor-bearing mice. Fluorescence imaging revealed strong signals localized at the tumor site within 2 h post injection (Fig. 6A). Following ultrasound application, the fluorescence intensity further increased, peaking at 4 h post-injection, with prominent signals observed not only at the tumor site but also in the peri-tumoral and distant lymph nodes, including the cervical LNs. Over the next 20 h, the fluorescence signal gradually diminished but remained detectable even after 24 h. *Ex vivo* fluorescence imaging further confirmed the significant accumulation of BI@PCM nanoparticles within tumor tissues and peritumoral LNs. Notable fluorescence signals were also observed in the liver and kidneys (Fig. 6A), suggesting that these organs play a role in the metabolism and clearance of nanoparticles. This delivery system not only facilitates the efficient transport of ETNs to LNs but also exhibits great potential to enhance immunotherapeutic efficacy by leveraging the immune activation capacity of ETNs within lymphatic tissues.

For therapeutic evaluation, K7M2 tumor-bearing mice were subjected to a 16-day treatment regimen, during which tumor size and weight were measured every other day. At the end of the treatment period, the mice were euthanized, and tumor tissues were harvested for further analysis (Fig. 6B). Throughout the study, no significant differences in body weight were observed across the treatment groups (Fig. 6D), and peripheral blood biochemical and hematological parameters of both short termed (1st days after the tumor was removed) and long termed (25th days after the tumor was removed) remained largely unchanged (Fig. 6C–SFig. 37), furthermore, according to the H&E results, no significant damage is observed in heart, liver, spleen, lung, and kidney tissues, throughout all treatment groups (SFig. 38), To assess the reproductive toxicity of this therapy, we also examined the ovarian tissues of mice from the BI@PCM and PBS groups using HE staining. The results indicated that there was no significant tissue damage in the ovaries of the BI@PCM group (SFig. 39). These results indicating the excellent biocompatibility of BI@PCM + US therapy. Given systemic indisulam administration and its potential for off-target splicing dysregulation, we quantitatively analyzed key splicing regulators beyond conventional biosafety assessments. Using ANAPC1—a ubiquitously expressed APC/C core subunit essential for ubiquitin-dependent proteolysis—as a sentinel gene, we observed no significant alterations in splicing ratios across major organs (SFig. 40), demonstrating tissue-sparing specificity. Additionally, to observe whether multiple injections trigger host antibody clearance, we assessed the antibody retention rate of Pd1 at different time points after several injections. The results showed that after the third and fifth injections, the decrease in Pd1 concentration was more pronounced than after fewer injections, though the difference was not significant (SFig. 41). This suggests that the impact of host antibody clearance is insufficient to affect the therapeutic efficacy. It is considered that it may be due to the long circulation time provided by the species homology of Pd1 and the cell membrane coating [50].

Compared to monotherapy with either BI@PCM or ultrasound (US) alone, combination treatment (BI@PCM + US) demonstrated markedly enhanced tumor suppression (Fig. 6E and F). Histological analysis using Hematoxylin-Eosin (H&E) staining of the tumor tissues collected on the final day of treatment revealed extensive tissue damage in the BI@PCM + US group, indicating robust antitumor efficacy (Fig. 6G). In contrast, other treatment groups exhibited significantly less tissue damage, underscoring the limited effectiveness of monotherapies. TUNEL staining further validated these findings by revealing the highest level of tumor cell apoptosis in the BI@PCM + US group (SFig. 42), highlighting the superior therapeutic potential of this combined approach.

### 2.7 Mechanistic study of immune cell activation and tumor microenvironment modulation by BI@PCM + US therapy

To elucidate the mechanisms underlying tumor suppression, we analyzed the expression of key markers, including Ki67, CRT, RBM39, and HIF-1, in tumor tissues using immunohistochemistry. These markers were selected to investigate the impact of the BI@PCM + US system on tumor proliferation, immunogenic cell death (ICD), disruption of alternative splicing, and hypoxia regulation. Compared to monotherapy with either BI@PCM or ultrasound (US) alone, BI@PCM + US treatment significantly downregulated the expression of Ki67 and HIF-1 (Fig. 6G), Pimonidazole staining further confirmed that BI@PCM + US potently alleviated tumor hypoxia, whereas monotherapies showed markedly less efficacy (SFig. 43). This finding aligns with HIF-1α downregulation, collectively proving ultrasound-triggered oxygen generation by BI@PCM. Furthermore, the BI@PCM + US group exhibited markedly elevated CRT protein levels compared to those of the other groups, providing strong evidence for the induction of robust ICD in tumor cells. Additionally, RBM39 expression was notably reduced in the BI@PCM + US, BI@PCM−US, and BI@CM + US groups, with the most pronounced decrease observed in the BI@PCM + US group. This finding highlights the ability of BI@PCM + US to efficiently promote RBM39 degradation in tumor tissues, consistent with its demonstrated efficacy *in vitro*. Collectively, these results underscore the multifaceted antitumor effects of the BI@PCM + US system, including tumor growth suppression, hypoxia mitigation, and the activation of immune-stimulatory pathways.

**Fig. 7:**
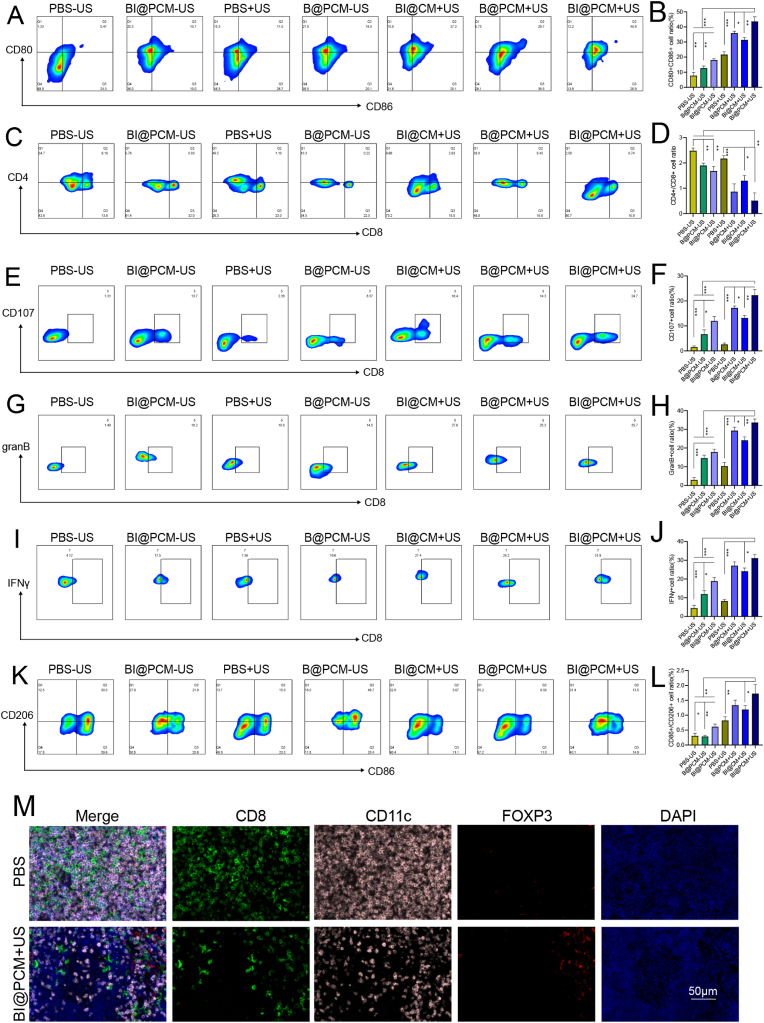
legend Mechanistic Insights into BI@PCM + US-Induced Immune Activation A. FACS plots of the DCs (gated by CD80+CD86^+^). B. Quantitative analyses of the DCs. C. Representative FACS plots of the CD8+/CD4+ T cells (gated by CD3). D. Quantitative analyses of the CD8+/CD4+ T cells. E. Representative FACS plots of the CD107+ T cells (gated by CD3, CD8). F. Quantitative analyses of the CD3^+^CD8^+^CD107+ T cells. G. Representative FACS plots of the granB + T cells (gated by CD3, CD8). H. Quantitative analyses of the CD3^+^CD8+granB + T cells. I. Representative FACS plots of the IFNγ+ T cells (gated by CD3, CD8). J. Quantitative analyses of the CD3^+^CD8+IFNγ+ T cells. K. Representative FACS plots of the M2/M1 TAMs (gated by CD86/CD206). L. Quantitative analyses of the M2/M1 TAMs. M. Multicolor immunofluorescence staining of peri-tumoral lymph nodes in the BI@PCM + US group and the PBS group.

To further investigate the immune responses elicited by BI@PCM + US treatment, we used ELISA and flow cytometry to analyze the tumor tissues. ELISA results revealed significantly elevated levels of key pro-inflammatory and immunogenic cytokines, including HMGB1, IL-1β, IL-18, and TNF-α, in the BI@PCM + US group compared to those in the control and untreated groups (SFigs. 44–47). These findings suggest that BI@PCM + US effectively enhances tumor immunogenicity and stimulates robust immune responses. Flow cytometry analysis demonstrated a marked increase in activated dendritic cells (DCs, CD80 [CD86], Fig. 7A and B) and a significant increase in CD8 + T cell infiltration, as reflected by the reduced CD4/CD8 ratio in the BI@PCM + US group compared to that of the other treatment groups (Fig. 7C and D). Further characterization of the CD8^+^ T cell subpopulation in Fig. 7E–J revealed that BI@PCM + US treatment induced the highest proportions of Granzyme B (GranB), IFN-γ, and CD107^+^-positive cells, indicative of strong activation of cytotoxic T lymphocytes (CTLs). Collectively, these results highlight the ability of BI@PCM + US combination therapy to effectively modulate the tumor immune microenvironment and activate adaptive immune responses, particularly through CTL activation. Furthermore, to further characterize T cell activation and demonstrate the role of PD1, IFN-γ and IL-2 ELISA assays were conducted (SFigs. 48 and 49). BI@PCM + US (high PD1) versus BI@CM + US (low PD1) confirmed that the PD1 membrane coating is indispensable for checkpoint blockade, leading to a significant increase in the concentrations of IFN-γand IL-2 in the BI@PCM + US. This validates PD1/PD-L1 targeting as the primary mechanism for immune reprogramming.

Tumor-associated macrophages (TAMs) that play a complex and dual role in the tumor immune cycle have also been investigated. M1-TAMs promote antitumor immunity by enhancing antigen release, antigen presentation, and T-cell activation, whereas M2-TAMs contribute to tumor progression by suppressing immune activation, supporting tumor growth, and facilitating immune evasion. Polarization analysis revealed that the M2/M1 ratio was significantly reduced in the BI@PCM + US group (Fig. 7K and L), indicating a favorable reprogramming of TAMs toward an antitumor phenotype.

To further evaluate the immune cycle, multicolor immunofluorescence staining was performed on the peritumoral lymph nodes from both the BI@PCM + US and PBS groups to assess immune cell activation. The results in Fig. 7M demonstrated an increased infiltration of dendritic cells (DCs, marked by CD11c positivity) and cytotoxic T lymphocytes (CTLs, marked by CD8 positivity) in the BI@PCM + US group, along with a notable reduction in the infiltration of regulatory T cells (Tregs, marked by Foxp3 positivity). These observations underscore the capacity of the BI@PCM + US system to enhance the tumor-immunity cycle at multiple stages, effectively promoting antitumor immunity and potentially improving the efficacy of immune checkpoint blockade (ICB) therapy. Additionally, the activation of long-term immune memory effects serves as another key indicator for evaluating the enhancement of the tumor immune cycle mediated by this therapy. To assess this, we also examined the percentage of effector memory T cells (Tem, marked as CD62L positive and CD44 negative) in the mice from each treatment group. The results indicated that the percentage of Tem cells was significantly increased in the BI@PCM + US group (SFigs. 50 and 51), confirming the enhancement of long-term immune memory effects by BI@PCM + US treatment. To further address this issue, we assessed the reactivation potential by measuring Ki67 expression in CD8^+^ T cells after co-culture with tumor antigens. Results demonstrated that T cells from BI@PCM + US-treated group exhibited significantly higher Ki67+ proliferation compared to PBS controls (SFigs. 52 and 53), confirming that memory T cells retain robust proliferative capacity upon antigen re-exposure. Furthermore, to evaluate whether the long-term immune memory contributed to tumor suppression, we observed the tumor recurrence response. The results showed that the tumor recurrence time was significantly delayed in the BI@PCM + US group, suggesting that its inhibitory effect on tumor recurrence was highly effective (SFig. 54).

## 3 Conclusion

**Scheme 2:**
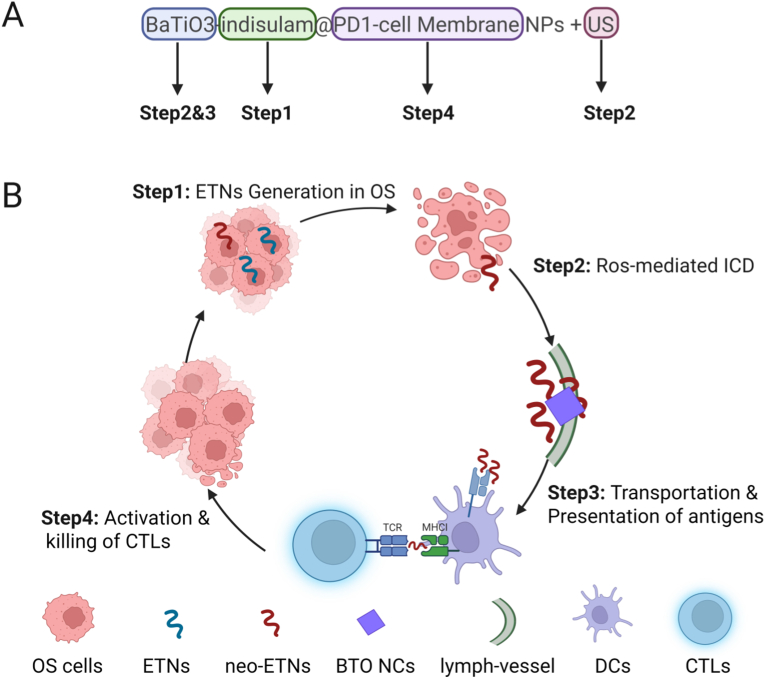

This study introduced and validated a novel nanoparticle system, BI@PCM, designed to comprehensively enhance the tumor immunity cycle (Scheme 2). By employing Pd1 cell membrane modification, the system achieved precise tumor targeting, whereas the ultrasound-triggered release induced immunogenic cell death (ICD) in tumor cells, significantly improving the generation and delivery efficiency of ETNs. The results demonstrate that BI@PCM combined with ultrasound bypasses traditional therapies that product tumor neoantigens through upregulating DNA damage pathways, to promotes the production of highly reactive neoantigens through the disruption of alternative splicing. Furthermore, the system enhances antigen presentation and T-cell activation by targeting the lymph nodes, effectively strengthening the key nodes of the immune cycle. In preclinical models, including organoids with immune microenvironments and tumor-bearing mice, BI@PCM + US exhibited exceptional immune activation and antitumor efficacy. This approach not only alleviates tumor hypoxia but also activates critical effector cells such as dendritic cells (DCs) and cytotoxic T lymphocytes (CTLs). Importantly, this strategy not only overcomes tumor immune evasion, but also provides robust support for personalized tumor immunotherapy, demonstrating substantial potential for clinical translation.

This study holds potential for complementing and enhancing the efficacy of CAR-T therapies. Specifically, following treatment with this therapy, personalized sequencing of patients can be used to design CAR-T cells specific to new antigens generated through alternative splicing, thereby further enhancing therapeutic efficacy. Alternatively, this therapy could be combined with currently developing CAR-T therapies activated by ultrasound [51]. Future studies incorporating mass spectrometry-based immunopeptidomics will be valuable to provide a direct molecular characterization of the MHC-I-bound neoepitopes generated by alternative splicing disruption. Future studies will incorporate rechallenge models to directly assess resistance to tumor re-implantation. In future designs, it may also be beneficial to introduce oncolytic viruses in a spatiotemporal coordinated combination therapy strategy. Oncolytic viruses can promote dendritic cell (DC) recruitment and maturation, thereby better enhancing the tumor immune cycle [52].

## 4 Materials and methods

### 4.1 Materials and instruments

Chemicals purchased from the Shanghai Chemical Corporation included Ba(NO_3_)_2_, tetrabutyl titanate (Ti [Bu]_4_), NaOH, KOH, hydrochloric acid (HCl), 1-butanol, oleylamine, oleic acid, toluene, ethanol, and ethylenediamine (DEA). TiO2 powder (anatase form) was obtained from Sigma-Aldrich. All the chemicals were used as received without additional purification. Deionized water was used in all experimental procedures.

### 4.2 Preparation of BTO

BaTiO3 nanocubes with an average size of 10 nm were synthesized using a hydrothermal method. In a 50 mL autoclave tube, 10 mL of an aqueous solution containing 2 mmol of Ba (NO_3_)_2_ and 10 mmol of NaOH was mixed with 3.6 mL of oleic acid (OA), 1.8 mL of oleylamine (OAm), and 20 mL of a 1-butanol solution containing 2 mmol of tetrabutyl titanate (Ti (Bu)_4_). The mixture was then agitated and sealed in an autoclave. The reaction was performed at 150 °C for 18 h. After the reaction, the system was allowed to cool to room temperature and the resulting products were isolated by centrifugation with ethanol. Finally, the collected nanocubes were dispersed in toluene to yield a stable milky colloidal solution.

### 4.3 Pd1 overexpression

To generate 293 T cells expressing Pd1, a lentiviral transduction approach was employed. The mouse Pd1 gene was first cloned into the lentiviral transfer plasmid [LV2 CMV-EF1α-copGFP(2 A)-Puro] to construct the Pd1 overexpression plasmid. The recombinant plasmid was then introduced into *Escherichia coli* (DH5α) for amplification, and this was followed by plasmid extraction. The Pd1 overexpression transfer plasmid, along with the packaging plasmids (pHelper 1.0 and pHelper 2.0), were co-transfected into 293 T cells using the Hieff Trans™ liposome transfection kit (Yeasen, 40802ES03). The cells were cultured in complete DMEM (Gibco, 11965-092) supplemented with 10 % fetal bovine serum (FBS, Gibco, 10099141). After 24 h of infection with a lentiviral suspension containing 8 μg/mL polyethyleneimine (H9268; Sigma-Aldrich), the cells were passaged into fresh culture medium. The success of transfection was assessed by evaluating the fluorescence intensity of the co-expressed green fluorescent protein (GFP) using an inverted fluorescence microscope (Nikon, TE2000) at 72–96 h post-infection. Cells that exhibited strong GFP fluorescence were successfully transfected. For further selection of genetically engineered 293 T-Pd1 cells with high purity and infection efficiency, puromycin selection was performed at an initial concentration of 1 μg/mL (Solarbio, P8230). The selected cells were reseeded for further expansion. Finally, 293 T-Pd1 cells were characterized and prepared for cell membrane extraction.

### 4.4 Preparation of BI@PCM

PD1-containing cell membranes were isolated from 293 T cells overexpressing Pd1 through a series of freeze-thaw cycles, and this was followed by ultracentrifugation. Purified cell membranes (20 mg) were combined with indisulam (6 mg) in a mixture of chloroform and methanol (5:1). After an incubation period of 1 h, 200 μL of chloroform-based BaTiO_3_ dispersion (1 mg/mL) was added to the solution. Subsequently, 2 mL of deionized water was added, and the mixture was stirred for 30 min to facilitate emulsification. To remove the organic solvent, the mixture was evaporated under reduced pressure, and 2 mL of deionized water was added. The suspension was incubated at 37 °C for 4 h to ensure the complete formation of the BI@PCM nanosuspension. The final product was stored in the dark at 4 °C for subsequent use.

### 4.5 Characterization of materials

The particle sizes, dispersion, and ζ-potentials of various NPs were analyzed using dynamic light scattering (DLS) on a Malvern Zetasizer Nano ZS90. Transmission electron microscopy (TEM; HT7700) was used to investigate the morphologies of the samples. The crystallographic structure of the as-prepared BaTiO_3_ was identified by powder X-ray diffraction (XRD) using a Shimadzu 7000 diffractometer equipped with a Cu Kα (0.15418 nm) radiation source. The size and morphology of the BaTiO_3_ nanoparticles were further examined using transmission electron microscopy (TEM, JEOL 2100) at an accelerating voltage of 200 kV. High-angle annular dark-field scanning transmission electron microscopy (HAADF-STEM) was conducted at 200 kV using a JEOL ARM200CF microscope that features a cold-field-emission electron gun for enhanced resolution. Raman spectroscopy was employed to characterize the vibrational modes of the samples using a Lab Ram ARAMISS system (Horiba Jobin-Yvon) with a 633 nm He-Ne laser as the excitation source. The chemical composition and functional groups of the BaTiO_3_ nanoparticles were analyzed by Fourier transform infrared (FTIR) spectroscopy using a Nicolet 6700 FTIR spectrometer. The piezoelectric properties of the BaTiO3 nanoparticles were assessed using atomic force microscopy (AFM; Cipher, Asylum Research). X-ray photoelectron spectroscopy (XPS) measurements were conducted using an ESCA-3 Mark II spectrometer (VG Scientific Ltd., UK), with Al Kα radiation (1486.6 eV). Binding energies were referenced to the C 1s peak at 285 eV, and measurements were performed with an accuracy of ±0.2 eV. Reactive oxygen species (ROS) production in the treated samples was detected using the fluorescent probe 2,7-dichlorodihydrofluorescein diacetate (DOJINDO) (Ex: 490–520 nm, Em: 510–540 nm). Additionally, dissolved oxygen levels were monitored using a dissolved oxygen meter (JPSJ-605 F) to assess the oxygen consumption during the experiments. Determination of Drug Loading were conducted by UV–Vis spectrophotometry (BRANSONIC M1800H equipment), Size exclusion chromatography (SEC) was performed to classify and quantify peptide segments and protein molecular weights (Coolaber). Sephadex G10 was used for the separation of peptides/proteins smaller than 0.7 kDa, Sephadex G15 for peptides/proteins in the range of 0.7–1.5 kDa, Sephadex G25 for proteins in the range of 1–5 kDa, and Sephadex G75 for proteins ranging from 3 to 80 kDa. For competitive binding assays between BTO NCs and ETNs, tumor cell lysates (containing ETNs) were pre-incubated with gradient concentrations (0, 50, 100, 200 μM) of spermine (a polycationic competitor) for 30 min. BTO NCs (100 μg/mL) were then added and incubated for 1 h. BTO-ETN complexes were isolated via centrifugation, and protein concentrations were quantified using the bicinchoninic acid (BCA) assay.

### 4.6 *In vitro* cytophagocytosis and cytotoxicity experiments

The cytophagocytosis of BI@PCM NPs was assessed using flow cytometry, and the fluorescence intensity of the CY5-labeled BI@PCM NPs was measured at various time points to track their uptake. Additionally, flow cytometry was performed to quantify the fluorescence intensity of the stained cells using a BD LSRFortessa instrument. To evaluate the *in vitro* cytotoxicity of the nanoparticles, K7M2 cells (2 × 10^5^ cells per well) were co-cultured with the NPs, and the medium was replaced with fresh culture medium containing 10 % CCK-8 reagent (Biosharp, Anhui, China). The cells were incubated for 1 h to allow for interaction with the reagents. Subsequently, cell viability was quantified by measuring absorbance at 450 nm using a microplate reader (Thermo Fisher Scientific, Inc., MA, USA).

### 4.7 Intracellular cell staining

Cell staining was performed by incubating the cells with various fluorescent probes. For the detection of reactive oxygen species (ROS), The DCFH-DA (MCE, Ex/Em = 488/525 nm) and HRF (MKbio, Ex/Em = 492/515 nm) probe was used to detect ROS. To assess the intracellular oxygen levels, [Ru (dpp)3]Cl_2_ (purchased from Maokang Biotechnology) was used with excitation at 488 nm and emission at 620 nm. To distinguish between viable and dead cells, a combination of calcein-AM (Beyotime) for live cell staining, with excitation at 490 nm and emission at 515 nm, and propidium iodide (PI) (Beyotime) for dead cell staining, with excitation at 530 nm and emission at 580 nm, was used.

Flow cytometry was used to quantify the infiltration of activitied DC cells (identified by CD86^+^ markers) and activitied lymphocytes (with CD69^+^ markers, detected using specific antibodies from BioLegend).

### 4.8 Transcriptome sequencing and proteomics

Comprehensive transcriptomic sequencing was performed to evaluate the changes in mRNA expression in osteosarcoma tissues following BI@PCM + US treatment. Total RNA was extracted from each tissue sample using the PureLink® RNA Kit (Invitrogen). Briefly, tissue samples were homogenized in lysis buffer containing 2-mercaptoethanol. After centrifugation, the resulting lysate was mixed with 70 % ethanol, and 700 μL of the solution was transferred to a spin column for further centrifugation. The column was subsequently washed with wash buffer, and this was followed by the application of RNase-free water and a final centrifugation step to elute RNA. Purified RNA was stored at −80 °C until sequencing was performed on the Illumina platform.

Raw sequencing data were processed and normalized using the RMA algorithm in the Transcriptome Analysis Console (Applied Biosystems). Gene-level expression data were derived from three independent RNA replicates for each experimental condition across three separate experiments. To analyze transcript alternative splicing variations, we employed the Hisat2, StringTie, and Astalavista packages, followed by visualization of transcript coverage using the ggcoverage package. Single-sample gene set enrichment analysis (ssGSEA) was conducted using the GSVA package to calculate hallmark gene set scores based on individual sample data.

The detection and analysis of alternative splicing events were performed using the SUPPA2 (v2.3) tool [53]. The specific steps are as follows: 1. Event Definition: Based on the GENCODE v44 reference annotation file, the SUPPA2 generateEvents command was used to generate definition files for various alternative splicing events. 2. Splicing Ratio Calculation: Using the transcript TPM value matrix, the SUPPA2 psiPerEvent command was applied to calculate the splicing percentage (Percent Spliced In, PSI) for each sample. For biological replicates of the same sample, the --pool-replicates parameter was enabled to merge data for improved statistical robustness. Next, to ensure that the detected alternative splicing events are biologically meaningful and statistically reliable, the following hierarchical filters were applied: 1. Coverage Filter: Only events with valid PSI values (i.e., PSI ∈ [0,1]) in at least 80 % of the samples were retained. 3. Expression Threshold: The associated transcript TPM value must be ≥ 1 in at least one experimental group to exclude low-expression background noise. 3. Differential Splicing Screening: Significance criterion: Benjamini-Hochberg corrected FDR <0.05. Effect size threshold: The absolute value of the average ΔPSI between groups must be ≥ 0.1 (i.e., splicing ratio difference ≥10 %). Finally, the data were visualized.

The prepared peptides were first subjected to separation for 2 h using an UltiMate 3000 rapid separation liquid chromatography system (RSCL, Thermo Fisher Scientific) equipped with C18 material, following the procedure outlined previously [54]. Subsequently, the peptides were ionized via a nano-source interface and introduced into a QExactive Plus mass spectrometer (Thermo Fisher Scientific) operated in data-dependent positive mode. Tandem mass spectra were acquired as follows: initial survey scans were performed with a resolution of 140,000, an AGC target of 3,000,000, a maximum ion time of 80 ms, a scan range of 350–2000 *m*/*z*, and profile mode. Fragment spectra were then recorded after quadrupole isolation (2 *m*/*z* isolation window) of the top ten precursor peptides with 2- and 3-fold charge states, under the following conditions: resolution 17,500, AGC target 100,000, maximum ion time of 60 ms, scan range 200–2000 *m*/*z*, and centroid mode. Precursor ions that had already been fragmented were excluded from further fragmentation for 100 s. Mass spectrometric data analysis was performed using MaxQuant version 1.6.10.43 (Max Planck Institute for Biochemistry, Planegg, Germany), which was employed for peptide and protein identification and quantification, using standard parameters.

### 4.9 Molecular biological detection

#### 4.9.1 Quantitative real-time PCR (qPCR)

RNA was extracted from cells that underwent various treatments, and gene expression was quantified using a real-time quantitative PCR instrument (Bio-Rad). Relative gene expression was calculated using the 2−ΔΔCt method after normalization to the endogenous control gene Gapdh. Primer sequences are provided as below:

Mouse Anapc1 (AP):

5′-acgtggatgtgacttgtccc-3′

5′-ggccaagtggaagccatagt-3′;

Mouse Clk4(ES):

5′-aacagtgtcttccgatgcgt-3′

5′-tgctgggatgggtcctaaga-3′;

Mouse Mrto4 (RI):

5′-atgcccaaatccaagcgaga-3′

5′-acccaaacagcaggatacgg-3′;

Mouse Xbp1(u):

5′-CCCTCCAGAACATCTCCCCAT-3′

5′-GGTCCAAGTTGTCCAGAATGCC-3′;

Mouse Xbp1(s):

5′-GAGCTGGGCATCTCAAACCT-3′

5′-CCTCCCAGGAGTGGTCTGTA-3′;

Mouse Gapdh:

5′-ttcaccaccatggagaaggc-3′

5′-tgaagtcgcaggagacaacc-3′.

#### 4.9.2 Western blot

The relative expression levels of the proteins Caspase-8 (*Anti*-Caspase-8 antibody, Abcam), calreticulin (*anti*-calreticulin antibody, Abcam), and RBM39 (*Anti*-RBM39 antibody, Abcam) were determined by western blotting. GAPDH (*anti*-GAPDH antibody, Abcam) served as an internal loading control to standardize protein levels across samples.

#### 4.9.3 Enzyme-linked immunosorbent assay (ELISA)

The concentration of inflammatory cytokines, including TNFα, IL-18, IL-1β, and CCL2, was quantified using commercially available ELISA kits (Thermo Fisher: Mouse TNF-α ELISA Kit, Mouse IL-18 ELISA Kit, Mouse IL-1β ELISA Kit, Mouse HMGB1 ELISA Kit, Mouse IFNγ ELISA Kit, Mouse IL12 ELISA Kit, Mouse TNFα ELISA Kit, Mouse IL2 ELISA Kit, Mouse Pd1 ELISA Kit) following the manufacturer's protocols.

#### 4.9.4 Immunofluorescence

For immunofluorescence analysis, cells or tissues were fixed with 4 % paraformaldehyde (Beyotime, P0099) for 10 min and blocked with QuickBlock™ blocking buffer (Beyotime, P0260) for 10 min. Samples were incubated overnight at 4 °C with primary monoclonal antibodies that included Anti-CD8 (Abcam), Anti-CD11c (Abcam), and *Anti*-Foxp3 (Abcam). After washing with PBS, secondary staining was performed using fluorescence-tagged goat anti-mouse IgG (H + L) (Beyotime, A0521) for 1 h at room temperature in the dark. The nuclei were counterstained with DAPI (Beyotime, C1006) for 10 min. After washing with PBS, the stained samples were analyzed by confocal laser scanning microscopy (CLSM, Leica, TCS SP8).

#### 4.9.5 Co-immunoprecipitation (Co-IP)

After culturing, the cells were frozen and homogenized, followed by the addition of RIPA lysis buffer to the ice-cold cell pellets. The supernatant was collected, and protein concentration was determined using the BCA method, then adjusted to 2–3 mg/mL. For each immunoprecipitation (IP) sample, a volume of 500 μL was used, with 30 μL reserved as input. Protein A/G Beads (50 μL of a 50 % bead concentration) were prepared and added to each 500 μL protein sample at 4 °C. The protein complexes were subsequently precipitated using magnetic bead-coupled antibodies targeting the bait protein. The protein complexes were separated by SDS-PAGE, and Western blotting was performed to detect the target protein.

### 4.10 Establishment of patient-derived osteosarcoma organoids and lymphocytes Co-culture models

Human osteosarcoma (OS) samples were obtained from patients who underwent their first osteosarcoma resection surgery between January and June 2024 at the Department of Orthopedics, Honghui Hospital, Xi'an, China (grant number: 202402001). The patient did not undergo any preoperative treatment. The study protocol was reviewed and approved by the Ethics Review Committee of Honghui Hospital and written informed consent was obtained from all patients.

The collected tissue samples were minced and treated with collagenase II (GIBCO). The resulting cell precipitate was filtered, combined with Matrigel (Corning), and cultured in an osteosarcoma organoid culture medium. This medium consisted of DMEM/F12 (GIBCO) supplemented with penicillin/streptomycin (Biological Industries), L-glutamine (STEMCELL), HEPES (GIBCO), B27 supplement (without vitamin A, GIBCO), N-acetyl-L-cysteine (Sigma), niacinamide (Sigma), recombinant human R-spondin-1 (Novoprotein), recombinant human epidermal growth factor (EGF) (Novoprotein), recombinant human fibroblast growth factor (FGF) (Novoprotein), Noggin (Novoprotein), A8301 (Abmole), and Y27632 (Abmole). After 7 days of culture, osteosarcoma patient-derived organoids (PDOs) were combined with autologous peripheral blood mononuclear cells (PBMCs) and allogeneic mesenchymal stem cells (MS) and further cultured in a matrix gel containing 30 % 1640 organoid culture medium.

### 4.11 *In vivo* antitumor therapeutic and immune function investigation

Animal Experimental Center of Peking University Medical School grant number: LA2019350. K7M2 tumor-bearing mice were divided into six experimental groups (n = 4) once the tumor size reached approximately 100 mm^3^. The (NPs) were intravenously injected into the tail vein. Tumor tissues were harvested at specific time points (1, 2, 4, 12, and 24 h post injection). To assess the biodistribution of the NP system, major organs (heart, liver, spleen, lung, and kidney) were collected 24 h after injection, and the distribution was analyzed using an in vivo imaging system (IVIS). Blood samples were collected from both the treated and control mice for biochemical analysis using the MNCHIP POINTCARE system and an automatic blood analyzer (MC-6200VET).

K7M2 tumor-bearing mice were randomly assigned to seven groups, including PBS, PBS + US, B@CM, B@CM + US, BI@CM + US, B@PCM + US, and BI@PCM + US. Mice in these groups received intravenous injections of 200 μL of either PBS or NPs five times, and this was followed by ultrasound (+US/-US) treatments. The treatments continued for 10 days. During this period, the tumor size and body weights were monitored every two days. The tumor volume (V) was calculated using formula V = L × W^2^/2, where L and W represent the longest and shortest tumor diameters, respectively. Two weeks after the initial treatment, the mice were euthanized, and the major organs (including the tumor, heart, liver, spleen, lung, ovary, and kidney) were collected for subsequent analysis. Hematoxylin and eosin (HE) staining was performed to examine the pathological status of the tissues, including damage to osteosarcoma cells. The expression of Ki67, Calreticulin (CRT), RBM39, and Hypoxia-Inducible Factor 1 (HIF1) in osteosarcoma tissues was analyzed using immunohistochemistry (IHC). Tumor infiltration was assessed using a Nikon imaging system and Image-Pro Plus software was used to quantify the ratio of positively stained areas within each field.

Flow cytometry was used to quantify the infiltration of dendritic cells (DCs) (identified by CD80+CD86^+^ markers), T cells (with CD4/CD8 ratios and cytotoxic T lymphocytes (CTLs) represented by CD107a, GranB, and IFN-γ), effector memory T cells (with CD62L positive and CD44 negative), and macrophage polarization markers. M1 (CD86) and M2 macrophage markers (CD206) were assessed to determine macrophage polarization in osteosarcoma tissues, all of the above immune cells were detected using specific antibodies from BioLegend.

As for tumor recurrence model, K7M2 xenograft tumor model was constructed to investigate the anti-tumor recurrence efficacy of NPs in vivo. When the tumor volume reached 150 mm^3^, it was surgically removed, leaving approximately 5 % of the residual tumor tissue to establish a postoperative tumor recurrence mouse model, recorded as day 0. On the seventh day post-surgery, the mice were randomly divided into seven groups (PBS, PBS + US, B@CM, B@CM + US, BI@CM + US, B@PCM + US, and BI@PCM + US) and use the same treatment plan as described previously, and the tumor recurrence of the mice was observed.

### 4.12 Statistical analysis

Statistical analyses were performed using SPSS (version 17.0; SPSS Inc., Chicago, IL, USA). Data are presented as mean ± standard deviation, derived from three independent experiments. For multiple group comparisons, one-way analysis of variance (ANOVA) was employed, followed by Dunnett's post-hoc test. In cases involving two independent variables, two-way ANOVA was used, with subsequent Bonferroni's post hoc analysis. Comparisons between two groups were conducted using Student's t-test.

## CRediT authorship contribution statement

**Linbang Wang:** Investigation, Conceptualization. **Yu Liu:** Methodology. **Ziyu Wang:** Investigation, Formal analysis. **Jingkun Liu:** Validation, Supervision. **Ziqiang Yan:** Validation. **Xiaoguang Liu:** Visualization, Validation, Funding acquisition.

## Declaration of competing interest

The authors declare that they have no known competing financial interests or personal relationships that could have appeared to influence the work reported in this paper.

## Data Availability

Data will be made available on request.
