# Supplementary material for: A comprehensive immune cycle enhancement strategy for alternative splicing-mediated endogenous Tumor neoantigens generation and delivery
Source: Mater Today Bio. 2025 Aug 30;34:102231. doi: 10.1016/j.mtbio.2025.102231 (PMC12448032; doi:10.1016/j.mtbio.2025.102231)

Supplementary material

SFigure 1 TEM image of BaTiO_3_ nanocubes. Scale bar, 10 nm


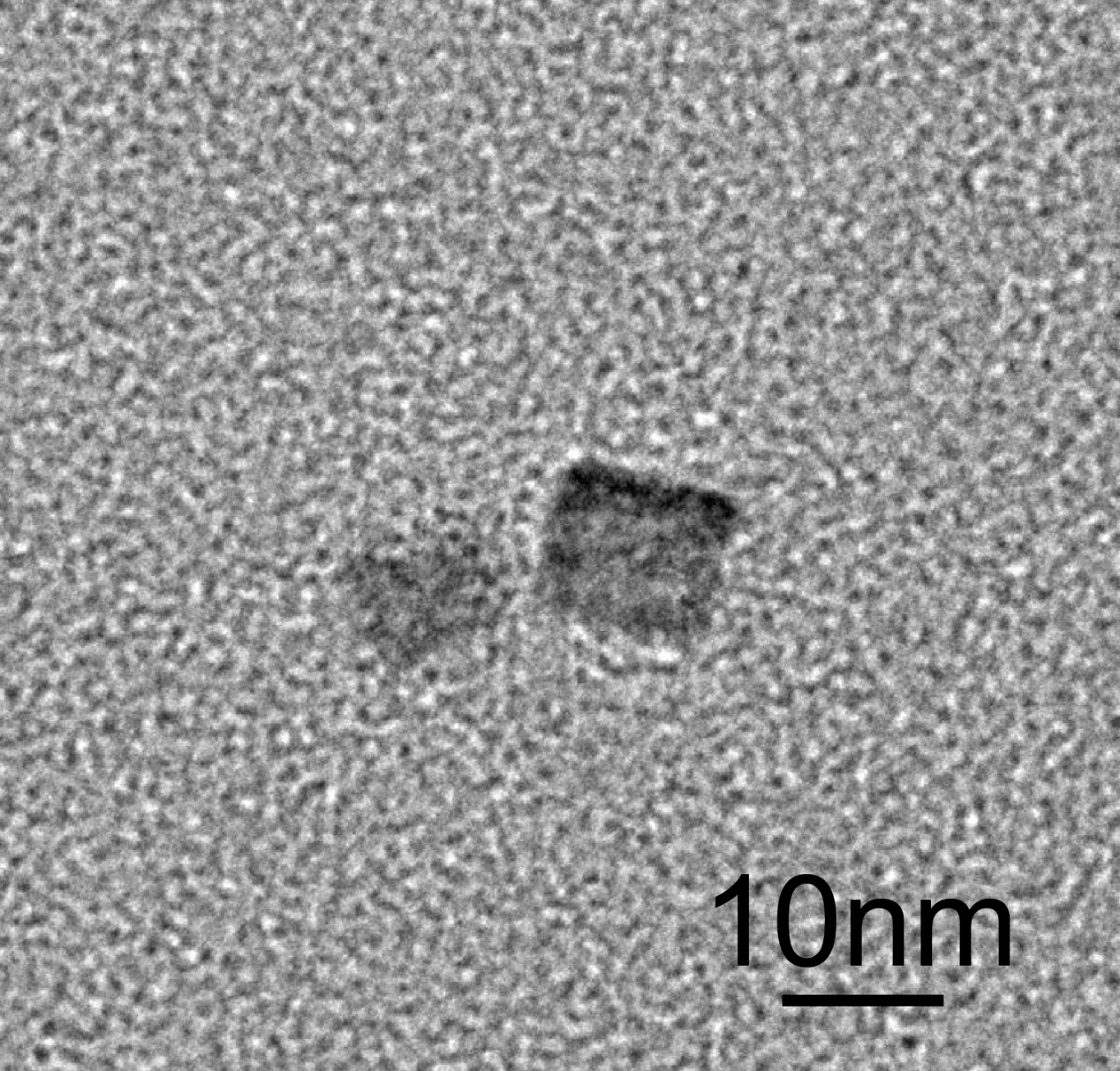


SFigure 2 Size and ζ potential changes of the BaTiO_3_ analyzed by DLS. (-) or (+) represented before or after incubating with US-treated K7M2 cells, respectively.

SFigure 3 Concentration-dependent changes of protein captured by BaTiO_3_ nanoparticles.

SFigure 4 Molecular mass ratio of tumor cell lysate (pre) and protein bound to BTO NCs (post)

SFigure 5 Spermine concentration dose-dependently inhibited protein adsorption on BTO NCs.​

SFigure 6 The fluorescence spectra of DCFH-DA under different conditions.

SFigure 7 O_2_ detection under different conditions.

SFigure 8 Schematic diagram of the plasmid skeleton


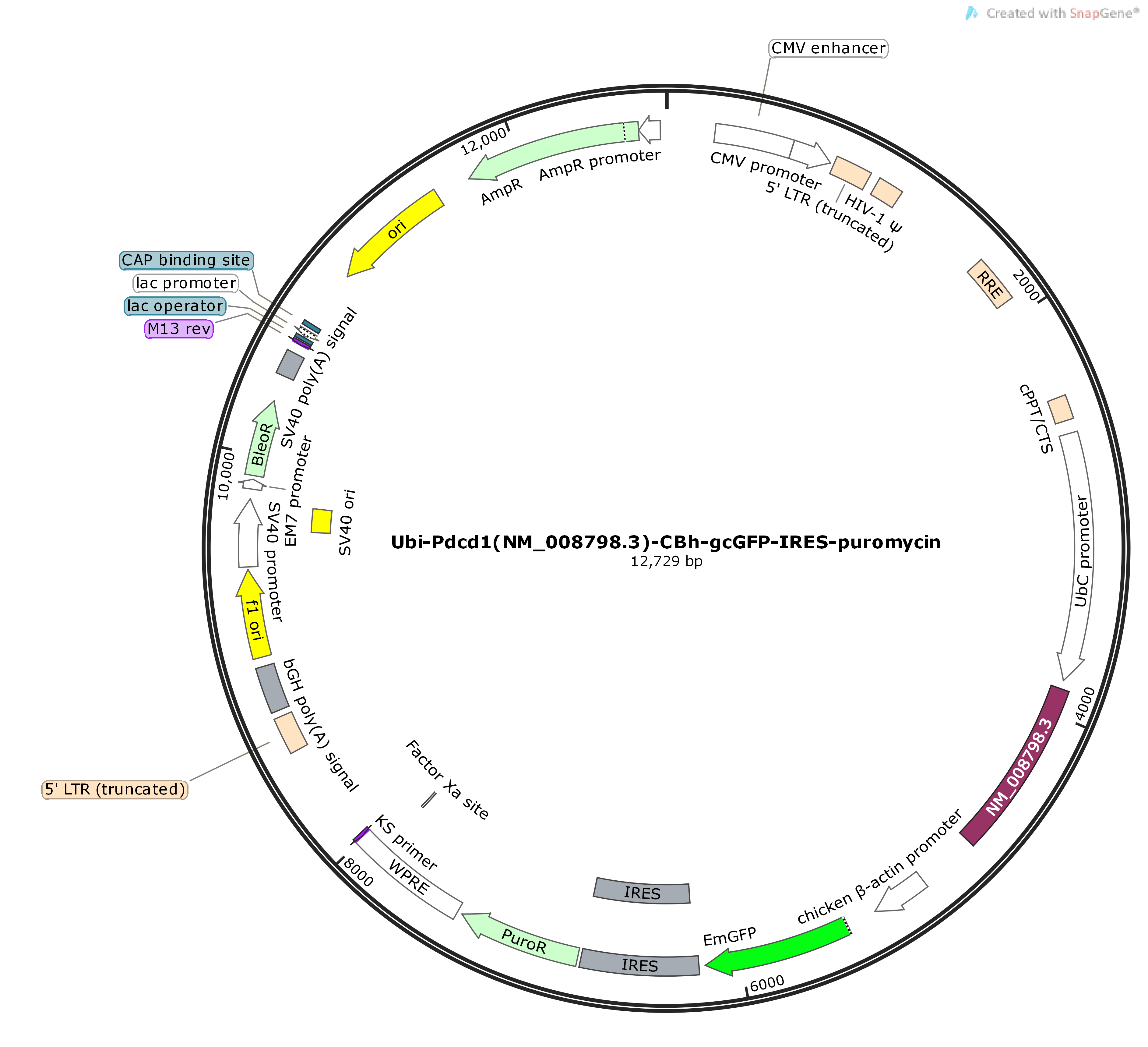


SFigure 9 Co-IP


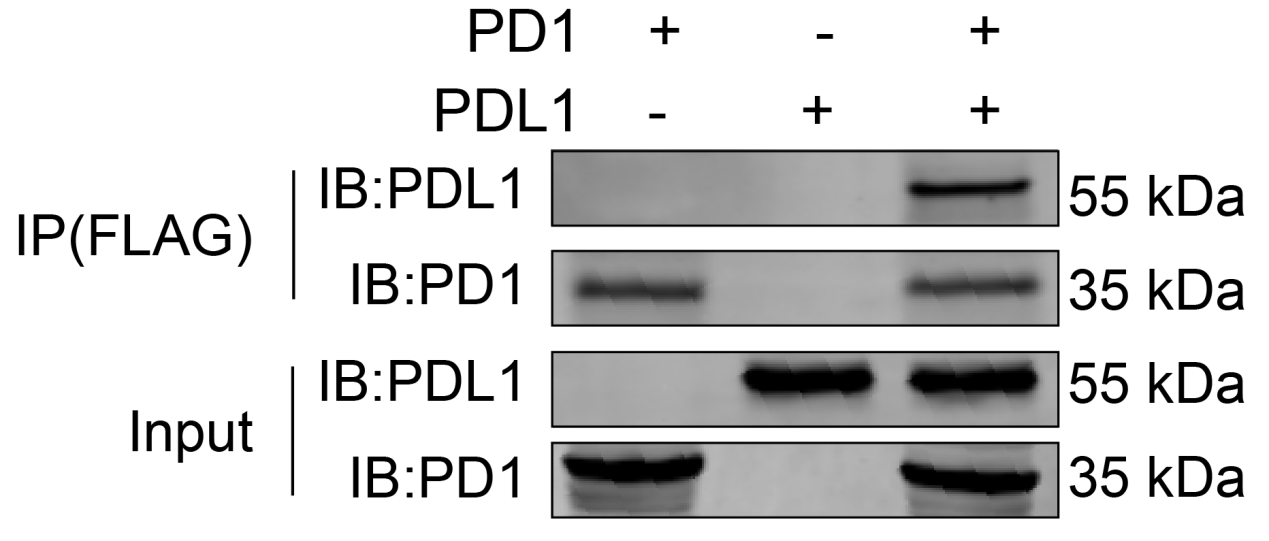


SFigure 10 FT-IR analysis of B, PCM, and B@PCM


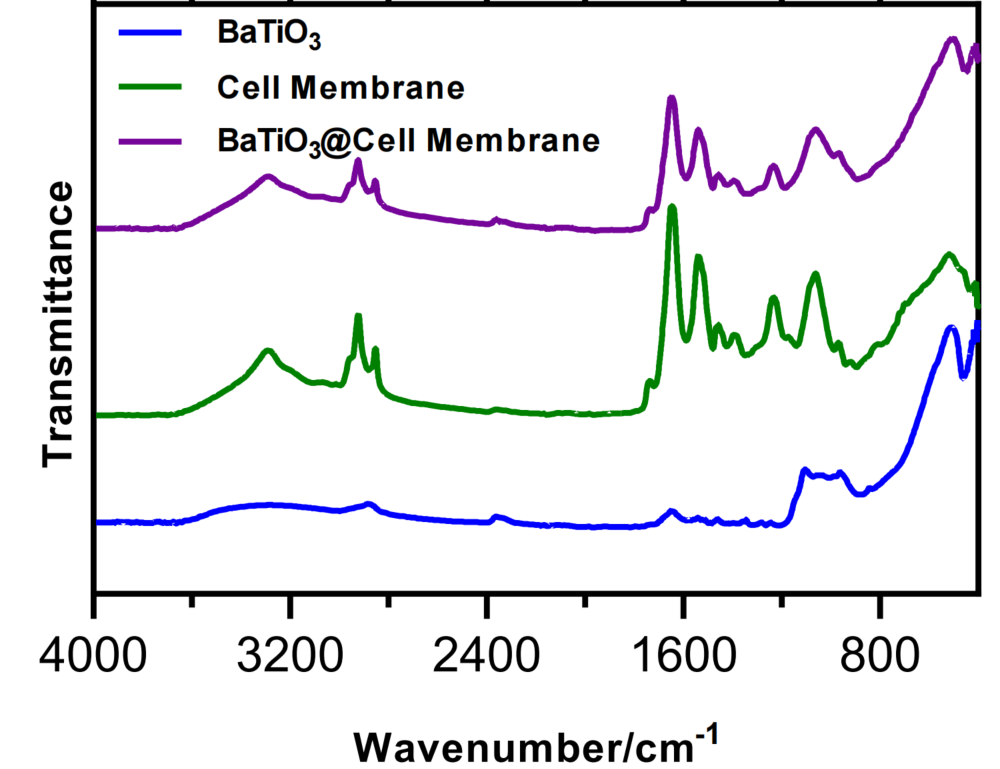


SFigure 11 UV-Vis analysis of BI@PCM, I, and B@PCM

SFigure 12 Cumulative remaining rate of the indisulam in BI@PCM

SFigure 13 The release of indisulam before and after US treatment

SFigure 14 The release of indisulam before and after US treatment under different power.

SFigure 15 Time-Resolved ROS Generation Dynamics of Membrane-Coated vs. Uncoated BTO NCs Under Ultrasound​

SFigure 16 Oxygen Evolution Kinetics of Membrane-Coated BTO NCs Under Piezoelectric Activation

SFigure 17 Flow cytometry of nanoparticle uptake in K7M2 cells after 4 h


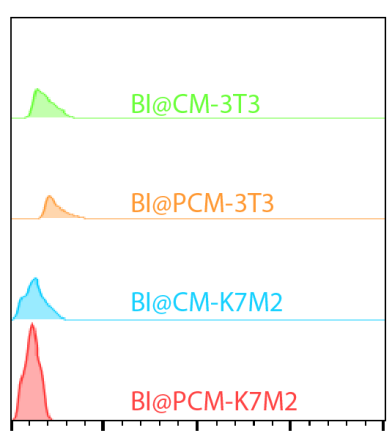


SFigure 18 Time-course quantification analysis of nanoparticle internalization in K7M2 cells measured by flow cytometry

SFigure 19 DCFH-DA fluorescence in cells across groups under the magnified field.


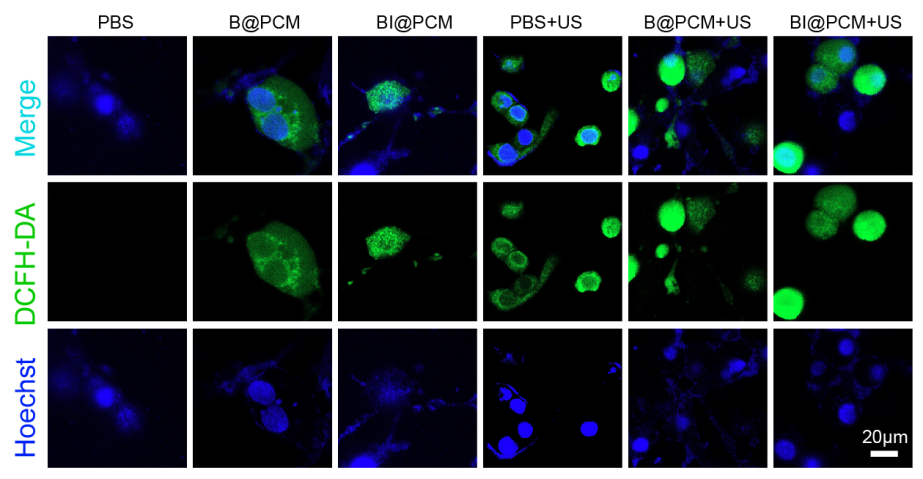


SFigure 20 Quantification of fluorescence intensity for DCFH-DA (n = 3, mean ± SD).

SFigure 21 HRF fluorescence in cells across groups under the magnified field.


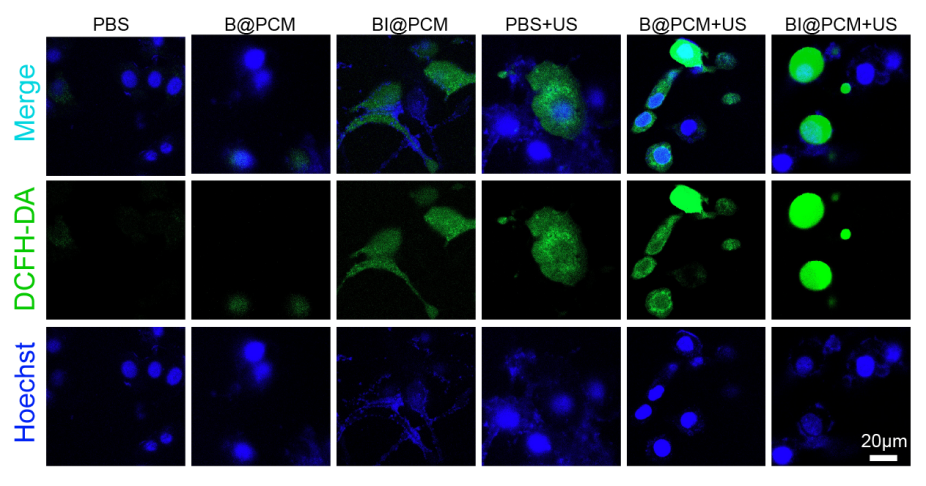


SFigure 22 Quantification of fluorescence intensity for HRF (n = 3, mean ± SD).

SFigure 23 Cell viability in terms of live/dead status across groups, including PBS-US, B@PCM-US, BI@PCM-US, PBS+US, B@PCM+US, and BI@PCM+US.


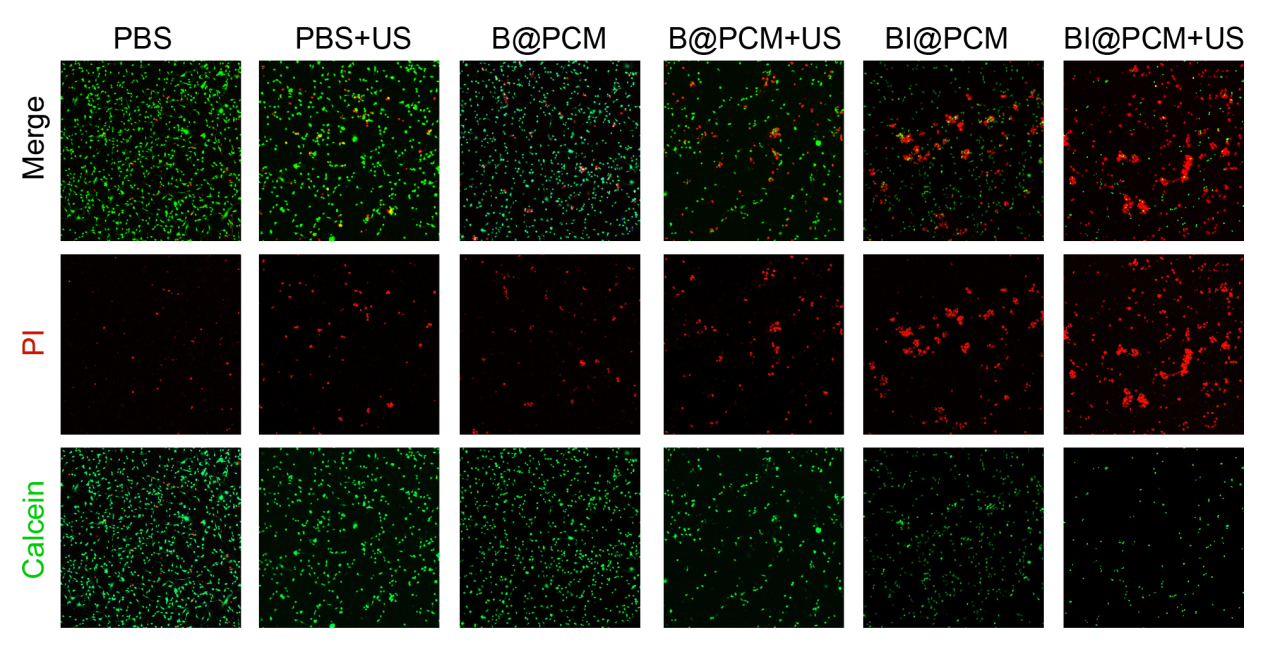


SFigure 24 Quantitative analysis of live/dead cell ratios across different groups.

SFigure 25 Quantification of HMGB1 by ELISA across the following groups, including PBS-US, B@PCM-US, BI@PCM-US, PBS+US, B@PCM+US, and BI@PCM+US.

SFigure 26 Volcano map of differential expression of alternative splicing subtype genes


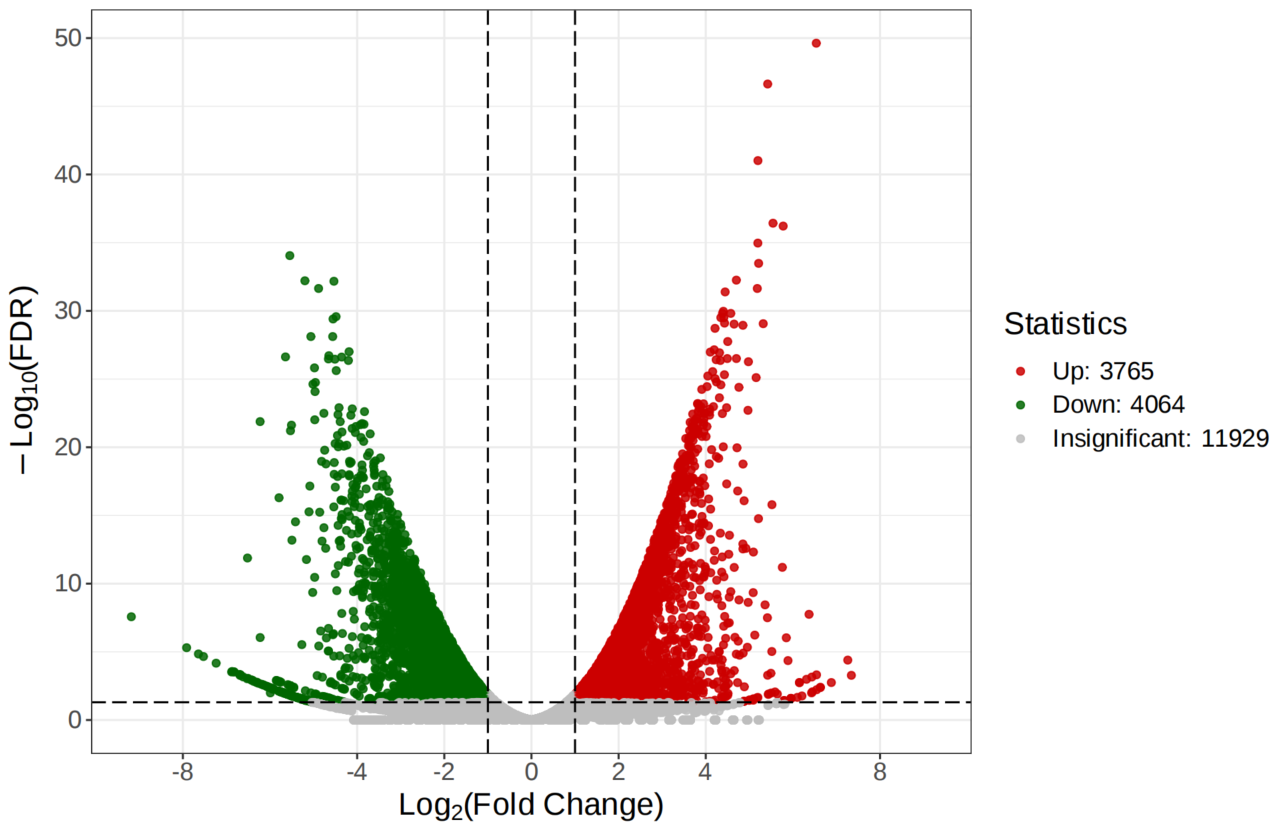


SFigure 27 Statistics of the number of variable splicing events before and after treatment.

SFigure 28 Quantitative and statistical analysis of key variable splicing events by RT-PCR

SFigure 29 Pie chart of subcellular localization of peptides

SFigure 30 Quantitative and statistical analysis of splicing variant mRNA XBP1(s) and XBP1(u) of XBP1 before and after treatment by RT-PCR

SFigure 31 Activation levels of DC cells after different treatments (CD86+ positive rate)
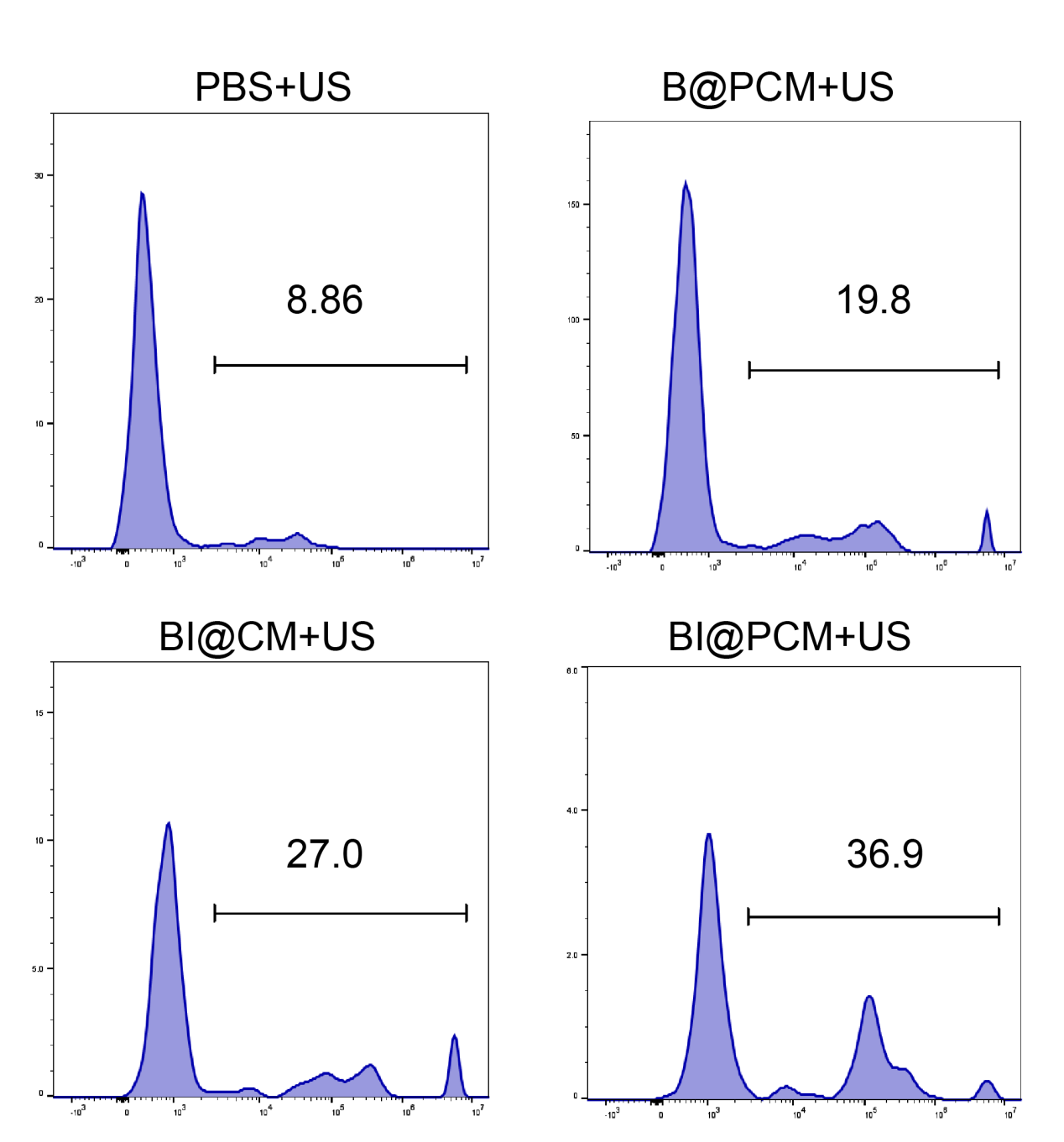
SFigure 32 Statistics of activation levels of Lymphocyte cells after different treatments (CD69+ positive rate)

SFigure 33 Statistics of DC cell activation level (IL-12 concentration) after different treatments

SFigure 34 Levels of lymphocyte activation after different treatments (CD69+ positive rate)


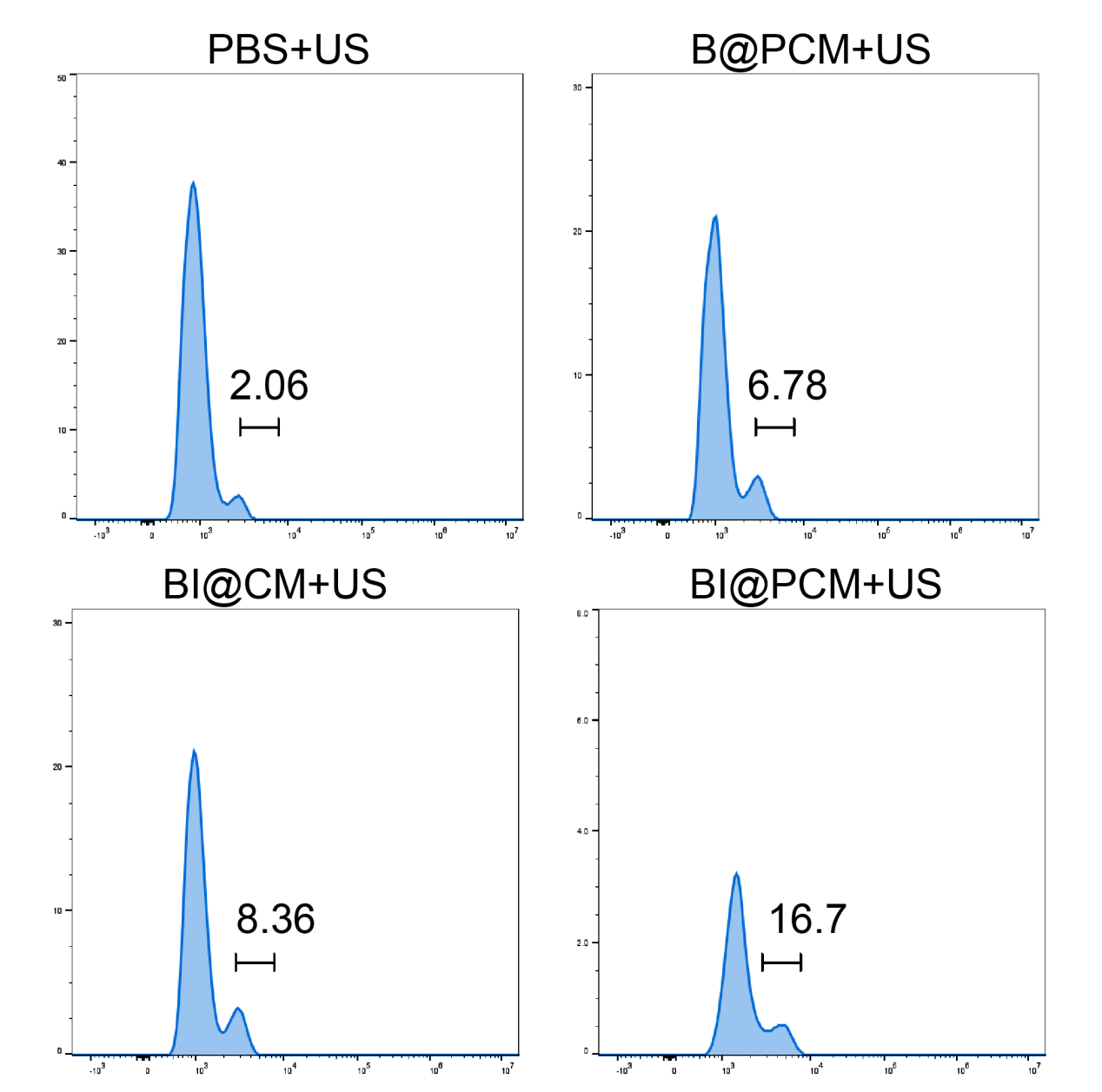


SFigure 35 Statistics of lymphocyte activation levels after different treatments.

SFigure 36 Statistics of lymphocyte activation levels (TNF-β) after different treatments

SFigure 37 Analysis of peripheral blood biochemistry and complete blood counts long-term post-treatment

SFigure 38 H&E images of the major organs after therapy across groups, including PBS-US, B@PCM-US, BI@PCM-US, PBS+US, B@PCM+US, and BI@PCM+US (scale bar: 100 μm).


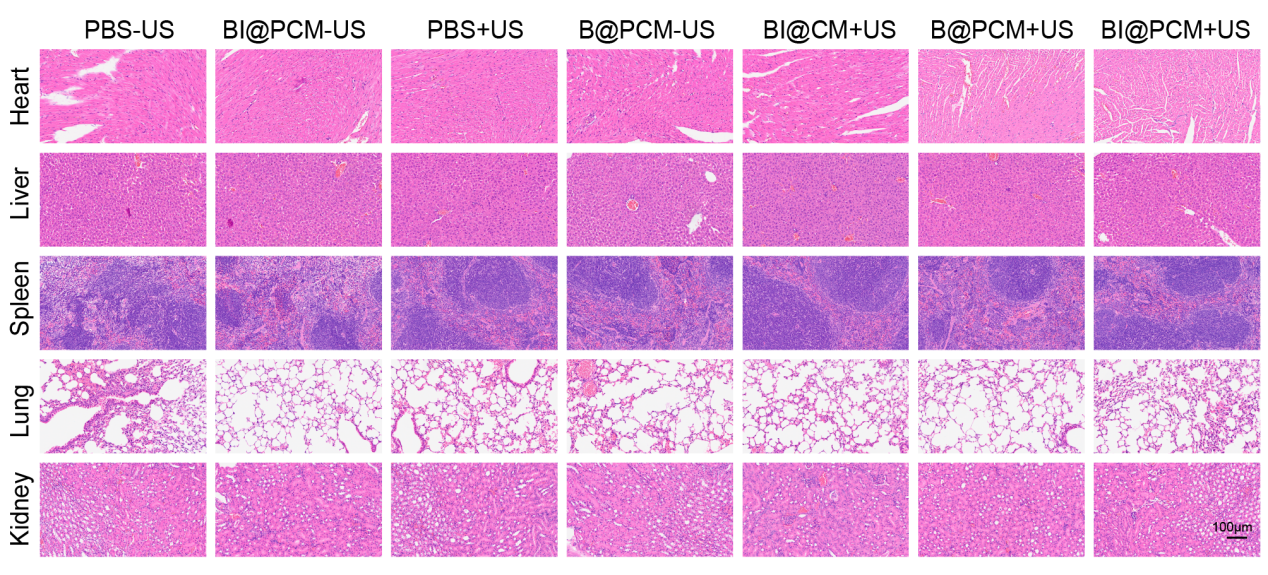


SFigure 39 H&E images of ovary


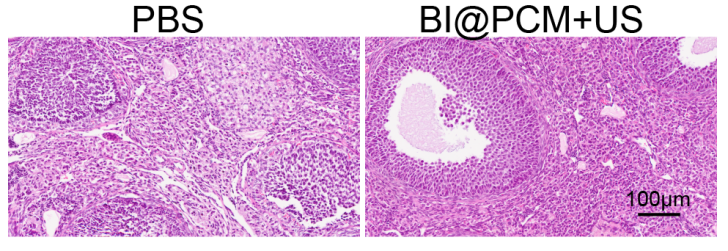


SFigure 40 Quantitative and statistical analysis of ANAPC1 gene AP splicing events of the major organsby RT-PCR

SFigure 41 Dynamic change of Pd1 residual level

SFigure 42 TUNEL staining of tumor tissues across groups, including PBS-US, B@PCM-US, BI@PCM-US, PBS+US, B@PCM+US, and BI@PCM+US (scale bar: 100 μm).


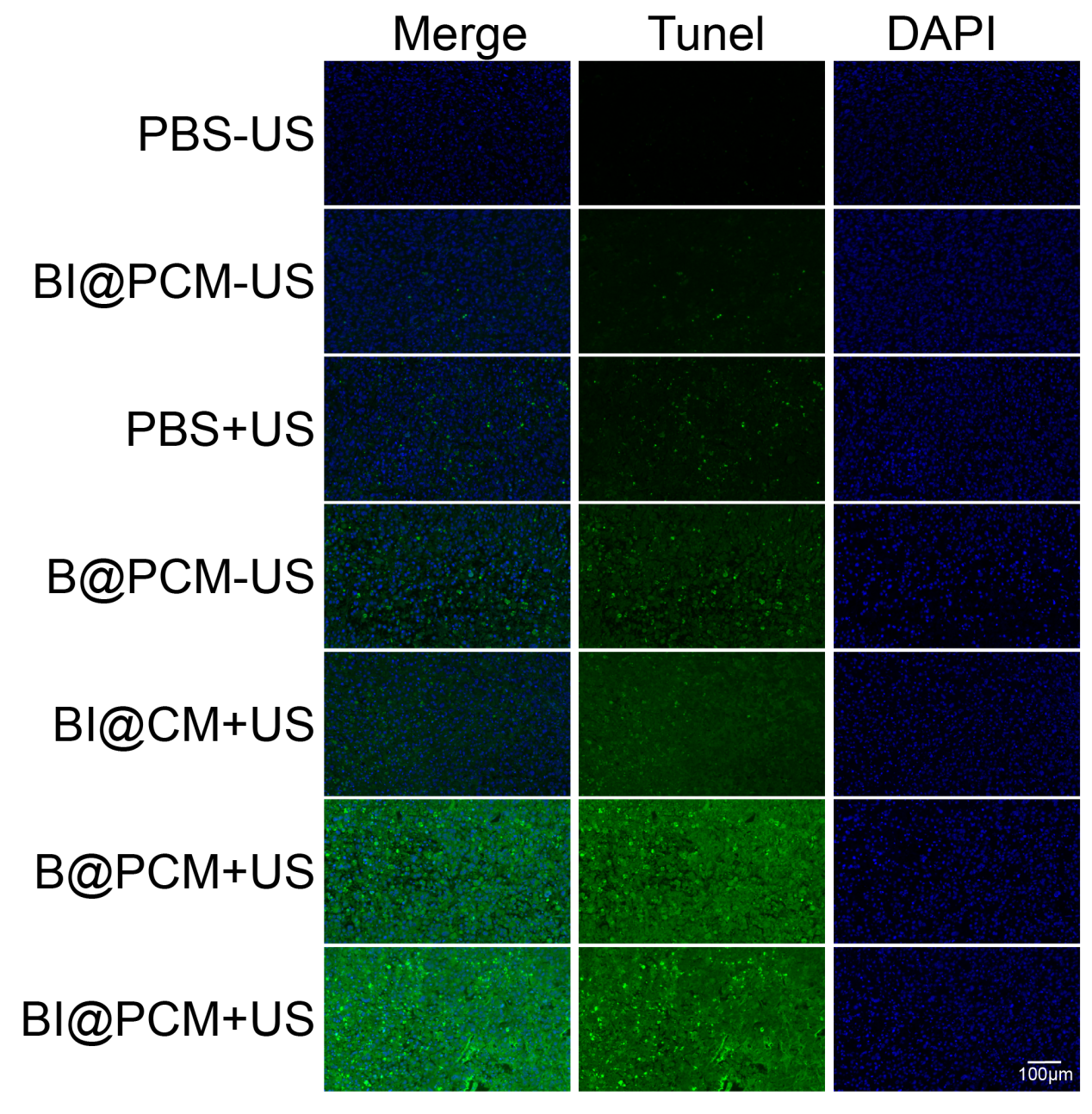


SFigure 43 Pimonidazole staining of tumor tissues across groups, including PBS-US, B@PCM-US, BI@PCM-US, PBS+US, B@PCM+US, and BI@PCM+US (scale bar: 100 μm).


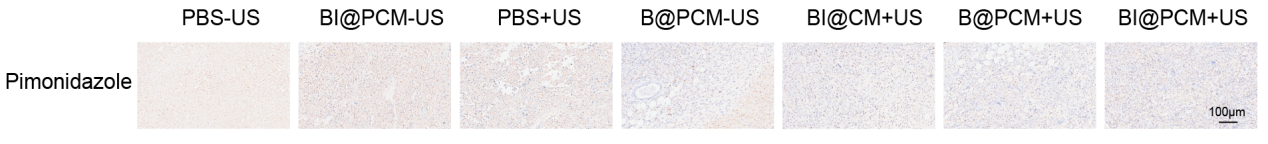


SFigure 44 Quantification of HMGB1 by ELISA across the tissues in following groups, including PBS-US, B@PCM-US, BI@PCM-US, PBS+US, B@PCM+US, BI@CM+US, and BI@PCM+US.

SFigure 45 Quantification of Il-1β by ELISA across the tissues in following groups, including PBS-US, B@PCM-US, BI@PCM-US, PBS+US, B@PCM+US, BI@CM+US, and BI@PCM+US.

SFigure 46 Quantification of Il18 by ELISA across the tissues in following groups, including PBS-US, B@PCM-US, BI@PCM-US, PBS+US, B@PCM+US, BI@CM+US, and BI@PCM+US.

SFigure 47 Quantification of Tnfα by ELISA across the tissues in following groups, including PBS-US, B@PCM-US, BI@PCM-US, PBS+US, B@PCM+US, BI@CM+US, and BI@PCM+US.

SFigure 48 Quantification of IFN-γ by ELISA across the tissues in following groups, including PBS-US, B@PCM-US, BI@PCM-US, PBS+US, B@PCM+US, BI@CM+US, and BI@PCM+US.

SFigure 49 Quantification of IL-2 by ELISA across the tissues in following groups, including PBS-US, B@PCM-US, BI@PCM-US, PBS+US, B@PCM+US, BI@CM+US, and BI@PCM+US.

SFigure 50 Representative FACS plots of the Effector memory T cells


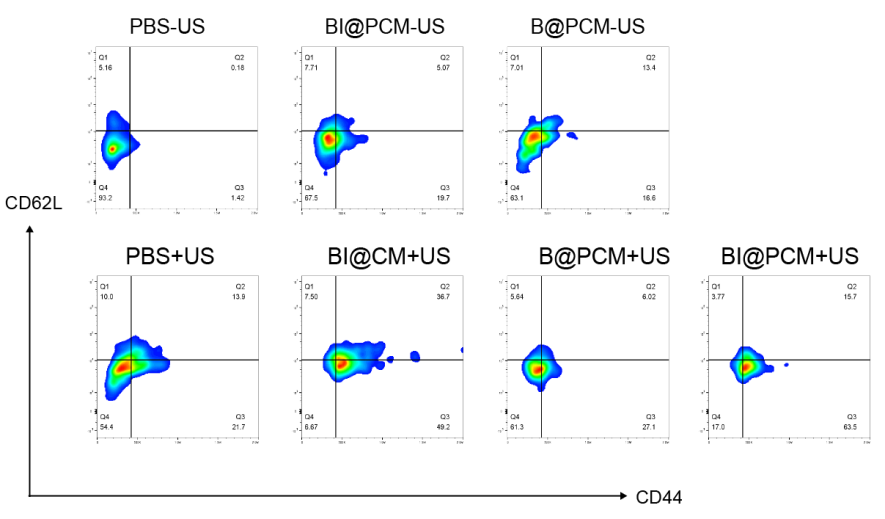


SFigure 51 Quantitative analyses of the Effector memory T cells

SFigure 52 Representative FACS plots of the Ki67+ Effector memory T cells


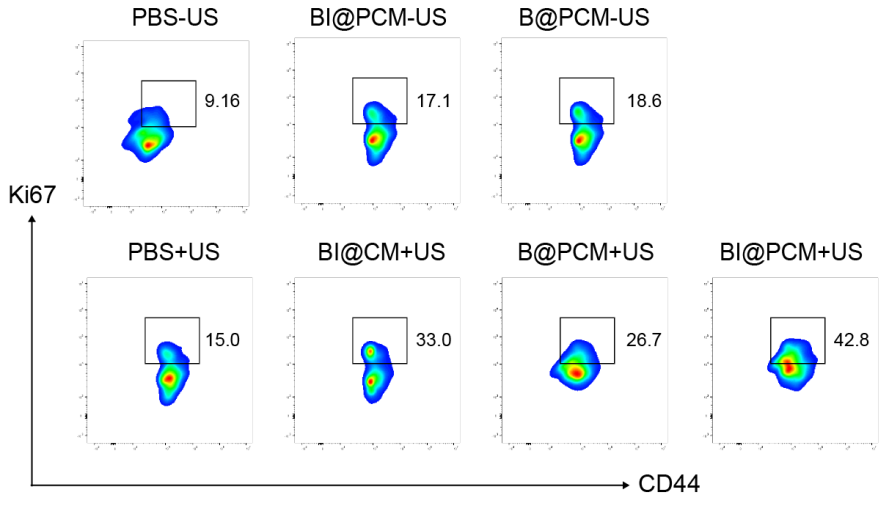


SFigure 53 Quantitative analyses of the Ki67+ Effector memory T cells

SFigure 54 Survival curve of tumor recurrence

Scheme 2 Comprehensive Enhancement of the Tumor-Immunity Cycle by BI@PCM+US. A. The composition of this therapeutic approach and the corresponding roles of each component in enhancing the tumor-immunity cycle. B. Demonstration of the tumor-immunity cycle process, specifically comprising four key stages.
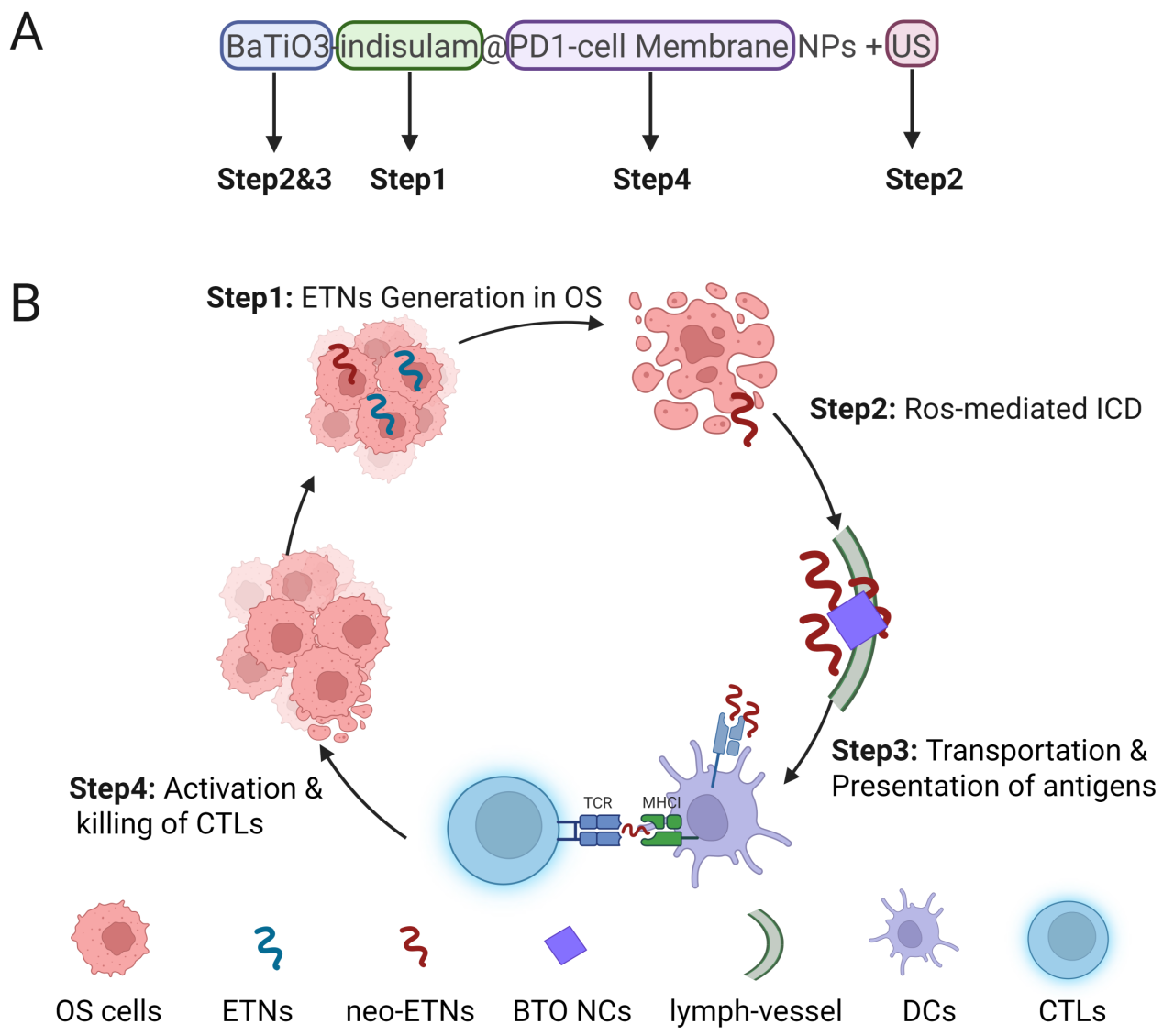

Supplement: Multimedia component 1 [file mmc1.docx]
